# Consumer Acceptance of Sustainable Dog Diets: A Survey of 2639 Dog Guardians

**DOI:** 10.3390/ani15202988

**Published:** 2025-10-15

**Authors:** Jenny L. Mace, Alexander Bauer, Andrew Knight, Billy Nicholles

**Affiliations:** 1 Centre for Ethics, Philosophy and Public Affairs, University of St Andrews, St Andrews KY16 9AL, UK; maceanimalwelfare@gmail.com; 2 Sustainable Pet Food Foundation, 147 Station Rd, London E4 6AG, UK; baueralexander@posteo.de (A.B.); billy@bryantresearch.co.uk (B.N.); 3 Mace Animal Welfare, Dunnock House, 63 Dunnock Road, Dunfermline KY11 8QE, UK; 4 School of Veterinary Medicine, College of Environmental and Life Sciences, Murdoch University, 90 South St., Murdoch, WA 6150, Australia; 5 School of Environment and Science, Griffith University, Nathan, QLD 4111, Australia; 6 Animal Welfare Research Group, University of Winchester, Sparkford Road, Winchester SO22 4NR, UK; 7 Bryant Research, 71-75 Shelton Street, Covent Garden, London WC2H 9JQ, UK

**Keywords:** dog welfare, dog diets, canine diets, vegan pet food, vegan dog food, sustainable dog food, cultivated meat-based dog food

## Abstract

Interest in more sustainable diets for the global population of 528 million companion dogs is steadily increasing, encompassing nutritionally sound cultivated meat, vegan, and microbial protein-based dog foods. Factors driving these alternative dog foods include lower impacts on the environment, fewer welfare problems related to intensively farmed animals and wild-caught fish, and potentially superior canine health outcomes, relative to conventional meat-based dog food. Through a questionnaire with 2639 responses, this study aimed to gain insights into dog guardians’ current feeding patterns and dog food purchasing determinants, acceptance of more sustainable dog diets, and sources of information used for decisions about dog diets. Key results included that 84% (2188/2596) of respondents currently fed either conventional or raw meat-based dog food. More than 43% (936/2169) of this group of respondents who answered found at least one of the more sustainable alternative dog foods acceptable, with purchases of these alternatives hinging most commonly upon the nutritional soundness of the products. Cultivated meat-based dog food was the most popular alternative (selected by 24%, 529/2169), followed by vegetarian (17%, 359/2169), insect-based (16%, 336/2169), and vegan (13%, 290/2169) dog food. The top three information sources used to make decisions regarding dog diets were labels/packaging (selected by 42% of all respondents, 1080/2596), scientific articles/books (38%, 989/2596), and business webpages (35%, 900/2596). Numerous human and dog demographic variables had impacts on current diets, acceptance of alternative diets, and information sources used. Notably, human diet and dog diet were the factors most commonly associated with current and potential purchasing decisions, as well as with information sources used. For instance, greater reductions by guardians in the consumption of animals were associated with greater acceptance of more sustainable dog diets. It should be noted that, due to the reliance on convenience sampling and the overrepresentation of respondents from the UK, of female guardians, of respondents with higher education, and of vegan guardians, the reported relative frequencies of subgroups were not fully representative of the global dog guardian population. Association estimates were based on regression analyses to minimize any resultant bias effects.

## Simple Summary

Guardians of the approximate 528 million companion dogs globally face an array of dog food choices. This is increasing with efforts to create dog food that involves less harm to other animals and a lower environmental impact, relative to conventional dog food. This survey of over 2600 dog guardians aimed to discern important factors guiding dog guardians’ dog food purchasing decisions—regarding both current diets and sustainable alternatives. We found that over 84% of respondents currently fed either conventional or raw meat-based dog food, but over 43% found at least one of the more sustainable alternative dog food options acceptable. Willingness to purchase the alternatives hinged most commonly upon the nutritional soundness of the products, with cultivated meat-based dog food proving the most popular alternative. Labels/packaging was the most frequently selected source of information used by guardians to make dog food decisions. Amongst the human and dog demographic variables analyzed, human diet and dog diet were the factors most commonly associated with current and potential purchasing decisions, as well as with information sources used. Our sample was not representative of the general population in certain respects. To minimize any resultant bias effects, we used regression analyses for the calculation of all association estimates.

## 1. Introduction

Dogs represent the most widely kept companion animal globally, with recent data estimating the worldwide companion dog population in 2024 at approximately 528 million, followed by companion cats at 476 million [1]. Other sources demonstrate similar population sizes [2]. The prevalence of companion dogs continues to increase internationally; for example, in Europe, there was an estimated 40% rise in households owning dogs between 2010 and 2022 [3], while China experienced an average annual growth of almost 10% in its pet dog population from 2018 to 2022 [4]. As a result, the global dog food industry was valued at over USD 41.4 billion in 2022, with projections indicating an annual growth rate of 5.1% through 2030 [5]. Despite this growth, concerns have recently emerged regarding the impact of traditional meat-based pet foods on (1) canine health, (2) environmental sustainability, and (3) the welfare of intensively farmed and wild-caught animals.

Health-related reservations primarily focus on the perceived low quality of some pet foods. These concerns arise from the incorporation of animal by-products (ABPs) sourced from slaughterhouses [6], the potential presence of harmful toxins [7], and the frequency of product recalls [8]. Additionally, the highly processed nature of many pet foods has raised concerns about nutrient depletion [9]. From an environmental perspective, the use of ABPs in conventional pet foods supports industrial animal agriculture, which is known to be a major driver of deforestation, habitat loss, water contamination, extensive land use, and climate change [10,11,12]. Notably, a review has indicated that pet food production in the United States accounts for 25–30% of the environmental impacts associated with animal agriculture overall [13,14]. Ethically, feeding dogs meat or fish necessitates the killing of other animals—unless cultivated meat-based dog food is utilized. Studies suggest that pet owners typically exhibit greater empathy towards animals and a stronger commitment to animal welfare compared to the general population [15], and there is a higher prevalence of vegetarians and vegans among pet owners [16]. This situation creates a moral inconsistency, as individuals who care deeply for their pets while feeding them meat-based diets may simultaneously contribute to the harm of other animals—a phenomenon described as the “vegetarian’s dilemma” [17] or the “animal lover’s paradox” [18]. Indeed, various theoretical frameworks, such as meat-related cognitive dissonance (MRCD), have been established to better understand how consumers make dietary purchasing decisions [19].

It is frequently argued that conventional meat-based dog food utilizes only the ABPs that are unfit for human consumption, thereby mitigating the worst of the welfare and environmental impacts [20]. However, Knight [10] has shown that nearly half (47.4%) of animal-derived ingredients in dog food sold in the US are actually suitable for human consumption, and that the remaining ABP fraction is associated with greater, rather than fewer, farmed animal welfare and environmental impacts when considering companion animal diets exclusively. Furthermore, there are indications that continued reliance on slaughterhouse ABPs for dog food could result in shortages, as ABPs are increasingly being diverted for renewable energy production [21]. In response to these health, environmental, farmed animal welfare, and ethical challenges, a growing array of alternative dog food products is now available.

### 1.1. Sustainable Alternatives to Conventional Meat-Based Dog Food

Nutritionally sound vegan dog food is the main genuinely sustainable option currently widely available for canine diets. While stakeholders frequently cite insect-based pet food as another sustainable alternative, unresolved questions regarding insect sentience [22] mean that welfare and ethical concerns associated with the use of farmed animals remain of concern. Furthermore, arable agriculture generally imposes a lower environmental burden compared to insect farming [23,24]. Other promising sustainable alternatives include dog foods produced from cultivated meat and fermented microbial proteins. The market for cultivated meat-based pet food is still in its infancy, with a limited batch of the first product becoming available in London, UK, in early 2025 [25]. Microbell represents the first commercially available dog food produced using fermented microbial proteins [26]. These emerging alternative pet food proteins have the potential to significantly reduce the environmental impacts associated with pet food consumption. However, regulatory barriers, such as unclear guidance and burdensome processes (particularly for small companies) remain, and need to be addressed [27]. Moreover, to tackle consumer concerns and misinformation surrounding these alternatives, some authors have urged governments and media organizations to run communication campaigns that inform guardians on the environmental and health impacts of different pet food types [28]. In contrast, the vegan dog food sector is already well established, valued at over USD17 billion and projected to exceed USD44 billion by 2032 [29]. Numerous companies currently offer nutritionally complete vegan dog foods (https://sustainablepetfood.info/suppliers, accessed on 7 October 2025), with South America leading the market and the highest anticipated growth in the Asia-Pacific region [29].

An expanding body of research is demonstrating the healthfulness of nutritionally complete vegan or vegetarian diets for dogs. As of September 2025, eleven studies had demonstrated good health outcomes associated with such diets [30,31,32,33,34,35,36,37,38,39,40], along with one systematic review [41]. Collectively, these twelve studies have reported no adverse effects—and sometimes health benefits. Veterinary and industry organizations are beginning to issue guidance on the safe transition to and maintenance of nutritionally sound vegan diets in dogs. For example, UK Pet Food [42] has released a fact and guidance sheet on vegan diets for dogs and cats, and the British Veterinary Association has issued advice on feeding dogs nutritionally complete vegan diets [43]. Both organizations support the use of these diets, when formulated to be nutritionally sound. Because of their relatively wide availability, and good dog health outcomes, this study focuses primarily on nutritionally sound vegan dog diets as the main alternative sustainable option.

Although recent scientific investigations into dog feeding practices and the factors influencing dog food purchasing decisions have advanced understanding of these aspects (e.g., [16,44,45,46,47]), they have provided limited insight into the willingness of dog guardians to adopt more sustainable diets, or the specific attributes required for these diets to be accepted. Such knowledge would enable the pet food industry to better address the diverse needs and preferences of different demographic groups of dog guardians and would assist veterinarians in advising their clients. Therefore, the present study was designed to investigate these additional aspects.

### 1.2. Research Questions

Three overarching topics were investigated: (1) existing feeding patterns and purchasing determinants among dog guardians; (2) acceptance by dog guardians of more sustainable dog diets; and (3) sources used by dog guardians to obtain information about dog diets. We analyzed these topics using the following specific research questions (RQs):

(1) Current diets:
  - What feeding patterns exist among dog guardians?
  - What factors do dog guardians find important when choosing dog diets?
(2) Alternative diets:
  - What proportion of dog guardians currently feeding meat-based (conventional or raw) dog food would realistically be willing to choose more sustainable alternatives?
  - For those willing, what characteristics would the alternative diets need to provide in order to be chosen?
(3) Information sources: Where do dog guardians source information about dog diets from?

## 2. Materials and Methods

### 2.1. Survey Design and Distribution

**Figure 1:**
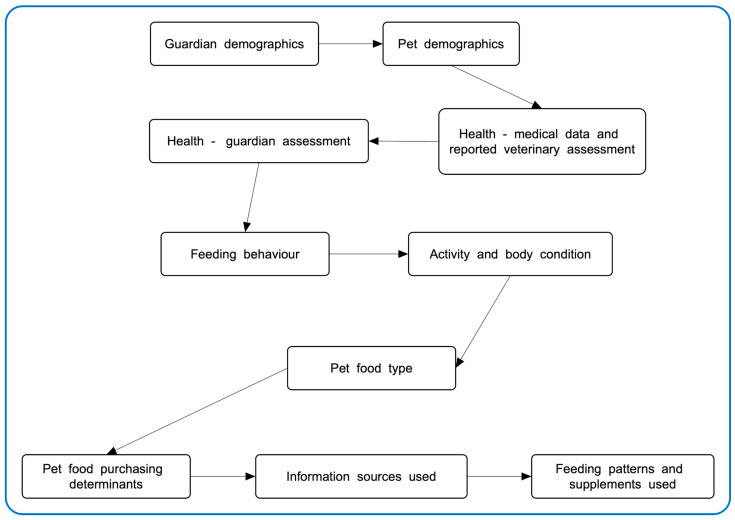
Sections of the questionnaire. Source: Knight et al. [37]. Note: The four sections connected to health, body condition, and behavior are not relevant to this study and so are excluded from this analysis; see Knight et al. [37,40,48] and Knight and Satchell [49] for analysis of these results.

Between May and December 2020, a quantitative questionnaire was administered via the Online Surveys platform (https://www.onlinesurveys.ac.uk, accessed on 14 October 2025) to gather data from dog guardians internationally regarding their current feeding practices, acceptance of more sustainable dog diets, and information sources used for dog diet decisions. After conducting a pilot study with 25 participants, the survey was revised—primarily by reordering questions—to reduce the risks of unconscious bias affecting responses. Specifically, items related to dependent variables were placed before those concerning independent variables, except for demographic questions, which remained at the beginning. The finalized survey instrument contained 37 principal questions organized into 10 sections, as illustrated in Figure 1. Depending on their responses, some participants received additional follow-up questions. The majority of questions were multiple-choice, frequently allowing respondents to select all applicable options, resulting in predominantly nominal data. The full survey is accessible on the OSF platform (https://osf.io/nbepu/, accessed on 14 October 2025).

Distribution of the survey occurred through social media channels, including specialized groups for dog enthusiasts. Paid Facebook advertisements were utilized to increase reach, and volunteers assisted in disseminating the survey. Targeted recruitment was employed for specific companion animal diet groups to ensure adequate sample sizes for statistical analysis. The choice of using Online Surveys as the survey platform was informed by its widespread adoption, with over 88% of UK universities, including our University of Winchester, using the platform in 2019 [50]. Participants within multi-dog households were instructed to select just one dog about whom to answer, and to base their responses on experiences from the preceding year. For households with both dogs and cats, respondents were asked to randomly select which animal to focus on, using the parity of their birth month as a guide (even-numbered months for dogs). Those whose dogs were on therapeutic diets were directed to reflect on their practices during the year before the therapeutic diet commenced.

### 2.2. Statistical Analysis

The four sections connected to health, body condition, and behavior were excluded from the analysis for the purpose of this study; see Knight et al. [37,40,48] and Knight and Satchell [49] for analysis of these results. Respondents not agreeing to the first screening question (n = 3) were removed from the data. The analysis of the remaining dog-based data is outlined in the following. For the analysis of the cat-based data, see a forthcoming study by Mace et al. [51].

Inferential statistics were utilized to analyze potential differences between both human and dog demographic subgroups and participant responses regarding RQs 1–3. Each research question was analyzed based on the estimation of multiple (generalized) linear regression models [52]. Linear regression models were estimated for (quasi-)metric response variables. Logistic regression models were estimated for binary response variables, as linear models are not suitable for such data [52]. All of the utilized category scores, outlined below, were created based on an intuitive grouping of the respective items.

For RQ1a, two logistic models were estimated on the binary questions “is the dog fed vegan or meat-based food?” and “is the dog fed raw meat or a conventional meat-based diet?” The former model was only estimated on the subgroup of vegan dog guardians because 303 out of 332 respondents feeding vegan dog diets were vegan themselves.

For RQ1b, four linear models were estimated on four quasi-metric category scores, each reflecting the share of individual items that each respondent stated were purchasing determinants for their current dog diet. The categories comprised Pet Focus I (with individual items ‘health and nutrition,’ ‘palatability,’ ‘diet quality’, i.e., pet-focused items directly influencing pet welfare), Pet Focus II (items ‘naturalness,’ ‘freshness,’ ‘diet reputation’, i.e., pet-focused items not directly influencing pet welfare), Personal Focus (items ‘price,’ ‘convenience,’ ‘social/cultural’), and Personal Values (items ‘food animals,’ ‘sustainability’). For RQ2a, six logistic models were estimated on the individual items ‘vegan,’ ‘fungi-based,’ ‘algae-based,’ ‘vegetarian,’ ‘cultivated meat,’ and ‘insect-based.’

For both RQ2b and RQ3, four logistic models were estimated on four category scores, each reflecting the information if at least one of the respective items was selected. The categories for RQ2b comprised Pet Focus I (items ‘health,’ ‘palatability,’ ‘quality,’ ‘nutritional soundness’), Pet Focus II (items ‘naturalness,’ ‘freshness,’ ‘reputation’), Personal Focus (items ‘price,’ ‘convenience,’ ‘social/cultural’), and Personal Values (items ‘sustainability,’ ‘animal welfare for cultivated meat,’ ‘animal rights for cultivated meat’). For RQ3, the categories comprised Product-Specific (items ‘label/packaging,’ ‘company webpage’), Vet/Pet Care (items ‘veterinarians,’ ‘other vet clinic staff,’ ‘pet store staff,’ ‘pet paraprofessionals’), Media/Literature (items ‘scientific literature,’ ‘media reports,’ ‘non-company webpage,’ ‘other books’), and Social Media (items ‘special interest group online,’ ‘general social media’).

All models controlled for the following sets of independent variables, with the reference characteristics indicated. Categories with a sufficient number of respondents and/or positioned at the start or end of ordinal variable categories were chosen as reference characteristics. Human demographic variables comprised the respondent’s dietary category (reference ‘omnivore’), categorized age (‘18–29’), gender (‘female’), education (‘doctorate’), potential occupation in the pet or veterinary industry (‘no’), income level (‘low’), geographical region (‘UK’), and type of residence (‘urban’). Dog demographic variables comprised the dog’s diet (‘conventional meat-based’), whether the dog was currently fed a medical diet (‘no’), age (‘0–4 years’), sex and neuter status (‘female, spayed’), breed size (‘toy’), if the dog was a working dog (‘no’), and his/her exercise level (‘normal’). As stated above, the human and dog diet effects were not estimated for all RQ1a models due to the high number of vegan-fed dogs with vegan guardians.

Human diet and dog diet were both substantially correlated with each other and with the other human demographic independent variables. To prevent this ‘multicollinearity’ from negatively affecting model estimations, the effects of human diet and dog diet were each estimated in separate regression models, which exclusively controlled for the additional dog characteristics. The resulting human and dog diet estimates are always reported side-by-side with all other estimates but are highlighted through gray-shaded areas in all figures to reflect this separated estimation scheme.

To account for the multitude of individual tests, multiple testing correction after Bonferroni-Holm [53] was applied individually for every model. Model assumptions were visually checked based on the distribution of model residuals. No relevant deviations from the assumptions could be observed. Additionally, we calculated the area under the curve (AUC) values for logistic regression models, which were calculated on a randomly selected 20% hold-out test set after re-estimating each model on the remaining 80% training set. The AUC values for RQ1a were 0.72 for the ‘vegan vs. meat-based’ model and 0.61 for the ‘conventional vs. raw meat’ model. Other AUC values ranged between 0.62 and 0.70 (RQ2a), 0.58 and 0.65 (RQ2b), and 0.61 and 0.66 (RQ3). The R^2^ values for the RQ1b linear regression models ranged from 0.02 to 0.06. AUC values below 0.7 are generally considered to indicate poor discrimination [54]. No such hard threshold is established for R^2^ values [55]. While some of our AUC and R^2^ values were very low, this was anticipated given our focus on modeling complex personal values and interests based almost exclusively on sociodemographic factors. In such social science research settings, low goodness-of-fit values do not necessarily indicate an uninterpretable model, but observed effect patterns and significances can and should still be interpreted [55].

Microsoft Excel was used to supply descriptive statistics. Inferential analyses were conducted using the open-source statistical software R version 4.5.0 [56]. Regression models were estimated with function “gam” from package “mgcv” version 1.9-1 [57]. AUC values were calculated with function “calc_auc” from package “plotROC” version 2.3.3 [58].

### 2.3. Research Ethics

This study adhered to the University of Winchester research ethics policy [59] and was approved under reference RKEEC200304_Knight. Prior to accessing the questionnaire, participants were provided with information regarding the study’s objectives, how the data they provide would be stored/used, the voluntary nature of any participation, who was carrying out the study, and contact details of who to contact regarding any ethical issues or remaining queries. The participants were then required to provide written confirmation that they were at least 18 years old and consented to participate, by ticking boxes to this effect. Additionally, respondents affirmed that they would answer questions based on one dog or cat they had cared for over the previous year. Full details can be found on the questionnaire, which, along with the dataset generated from this research, the mapping between dependent variables and questionnaire items, and the R code utilized for the statistical analyses, is publicly available at https://osf.io/nbepu (accessed on 14 October 2025). Perplexity (https://www.perplexity.ai) was used on 30 May 2025 to re-phrase parts of the introduction, methodology, and limitations sections of the paper. This was to avoid self-plagiarism of a related study by the same authors [51], and to prevent verbatim repetition between certain parts of the abstract and conclusions. The prompt used on Perplexity was “Can you re-write (paraphrase) in the same academic tone the following passage please? It’s to avoid self-plagiarism.” An example included changing this sentence in the introduction:

“The number of households with a dog continues to rise globally; for instance, across Europe, between 2010 and 2022, there was roughly a 40% increase in the number of households with a dog [3] and China’s pet dog population grew by 9.8% each year between 2018 and 2022 [4]”

to

“The prevalence of companion dogs continues to increase internationally; for example, in Europe, there was an estimated 40% rise in households owning dogs between 2010 and 2022 [3], while China experienced an average annual growth of almost 10% in its pet dog population from 2018 to 2022 [4]”.

## 3. Results

Data cleaning included the removal of respondents (a) not agreeing to the first screening question (n = 3), (b) stating they played no role in pet food decisions (n = 16), and (c) leaving all questions blank after the demographics section (n = 27). Following this, there were 2596 respondents. Additionally, for the regression-based analyses of associations of data with human and dog demographic characteristics, pregnant (n = 3) and lactating (n = 3) dogs were excluded, due to their non-standard nutritional requirements, leaving 2590 respondents. This section first describes the human and dog demographic characteristics of the remaining participants, before proceeding to explore the three key topics outlined in Section 1.2. Each subsection comprises descriptive statistics followed by the most important results from the regression modeling of associations between human/dog demographic characteristics and the variables of interest.

In reporting these results, we have applied the following conventions:

- Effect = significant association after multiple testing correction.
- Trend = significant association prior to multiple testing correction.
- No trend = no significant association prior to multiple testing correction.
- Explorative tendency = an association arising from explorative analyses only.
- Tendency = general pattern.

Only the most important results—effects and particularly noteworthy trends—are outlined below; comprehensive results are supplied within Supplementary Tables S1–S3.

### 3.1. Human and Dog Demographic Characteristics

In terms of dog guardian demographics, 91.6% (2377/2596) of respondents were female; 71.6% (1858/2596) were from the UK; 80.1% (2079/2596) had some experience of college-/university-level education; and 64.3% (1670/2596) stated they had a ‘Medium’ level of income relative to ‘Low’ (14.8%, 384/2596), ‘High’ (13.9%, 360/2596), and ‘Prefer not to answer’ (7.0%, 182/2596). There was a fairly even spread between the ages of 20 and 69, with the most represented age range being 50–59 (23.0%, 596/2596). Similarly, 35.7% (928/2596) lived in an urban environment, but around 32.0% lived in both rural and equally urban/rural areas (32.0%, 830/2596; 31.7%, 823/2596, respectively). Moreover, 40.2% (1044/2596) had a standard omnivore diet. Additionally, 81.4% of respondents (2114/2596) did *not* work in the pet care industry, as a veterinarian, veterinary nurse/technician, animal trainer, nor animal breeder. Table 1 summarizes additional human demographic information.

**Figure 2:**
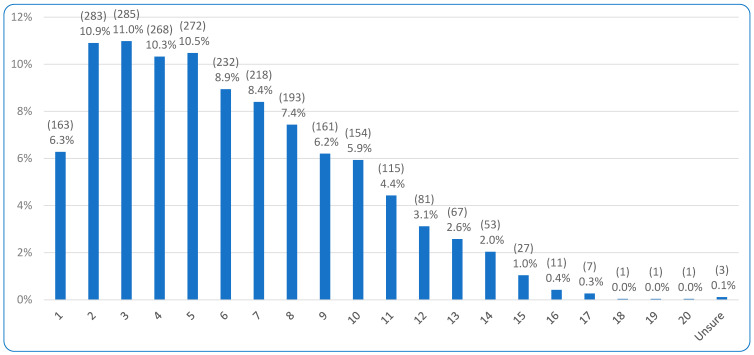
Distribution of dog ages (n = 2596).

In terms of dog demographics, 98.6% (2559/2596) stated their dog was a companion animal rather than working animal. Figure 2 demonstrates the age ranges of the animals; age 3 was the most common age, selected by 11.0% of respondents (285/2596), with ages 2, 4, and 5 also represented by over 10% of respondents. The vast majority of dogs were neutered (78.2%, 2028/2596), which equated to 81.8% of female dogs (1001/1224) and 74.9% of male dogs (1027/1372). Additionally, almost all dogs (95.9%, 2489/2596) did not have specific high energy requirements (e.g., high levels of exercise, lactating, or pregnant), and 95.2% (2472/2596) were not on a prescription diet. Indeed, 94.2% (2446/2596) of respondents stated their dog was either ‘Healthy’ (61.6%, 1600/2596) or ‘Generally healthy’ (32.6%, 846/2596). Additionally, there was a wide distribution of small (20.1%, 521/2596), medium (38.8%, 1008/2596), and large (34.2%, 889/2596) dogs, along with a few giant (4.4%, 113/2596) and toy (2.5%, 65/2596) dogs.

### 3.2. Current Feeding Patterns and Purchasing Determinants

#### 3.2.1. Current Feeding Patterns

**Figure 3:**
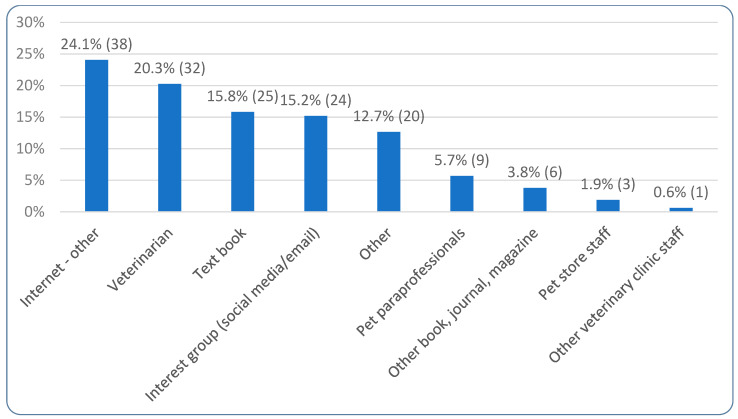
Distribution of sources of homemade dog food recipes (n = 158).

Table 2 demonstrates the diets primarily fed to the dogs, with ‘Meat-based—conventional’ comprising a majority at 52.3% (1359/2596), followed by raw meat-based diets (31.9%, 829/2596) and vegan diets (12.8%, 333/2596). Table 3 demonstrates where guardians obtain the majority of their dog food, with ‘Direct from manufacturer’ being selected most commonly by 25.6% (664/2596). Table 3 further highlights how commercial dog food comprised between 75% and 100% of the diet of nearly three quarters (71.1%, 1845/2596) of respondents’ dogs. It also highlights how the diets of respondents’ dogs were over 50% homemade in 9.8% (254/2596) of cases. Echoing this, the same table displays how up to 10.9% (285/2596) of respondents may be using homemade food as dog food, while ‘Commercial dry kibble’ is used most commonly by 37.6% (976/2596). When specifically asked if homemade food comprised more than half of their animals’ diets, 14.0% (364/2596) said ‘Yes.’ Of these 364 respondents, less than half (43.4%, 158/364) used a recipe, while 56.6% (206/364) did not. Figure 3 displays the sources for the recipes among those using a recipe, with ‘Internet’ being the most common (24.1%, 38/158).

‘Twice daily’ was the most common frequency of daily feeding (73.6%, 1910/2596), followed by ‘Once daily’ (11.9%, 310/2596), ‘Three times daily’ (8.2%, 213/2596), ‘Food is always available’ (4.3%, 112/2596), and ‘Other’ (2.0%, 51/2596). Common answers for people selecting ‘Other’ included 4–6 times a day and references to their dog ‘working’ for their food or no meals per se with food allowances instead being incorporated into training. Just under half (48.4%, 1256/2596) weighed the food given daily.

**Figure 4:**
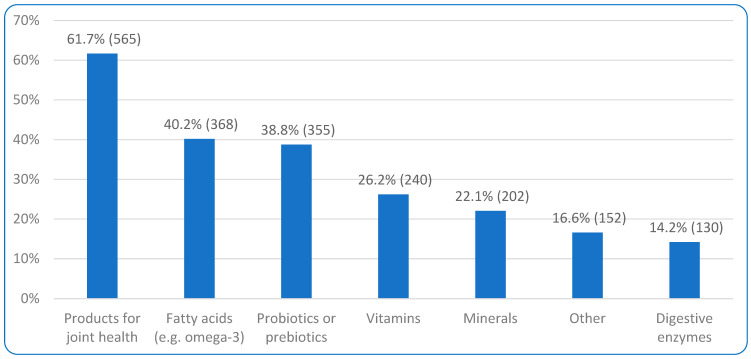
Distribution of dietary supplements given to dogs (n = 916). Note: Multiple responses were possible; thus, the percentages and counts represent the proportion of all respondents selecting each answer option.

Over 90% (91.3%, 2371/2596) fed treats/snacks/scraps to their dog. Table 4 displays the frequencies of these with ‘More than once a day’ being selected by almost half of the respondents (48.9%, 1160/2371). The most common treat given was ‘vegetables or fruit’ (56.6%, 1342/2371), as displayed in Table 4. Over 60% (63.9%, 1658/2596) did not give their dog any supplements. Of the 36.1% (938/2596) who did give supplements, 11.0% (103/938) gave amino acids. When asked which, common responses included YouMove Plus (Glucosamine), Vegedog supplement, incorporated into dog food bought, taurine, lysine, L-Carnitine, DL-methionine, and tryptophan. The other supplements given to respondents’ dogs are summarized in Figure 4.

**Figure 5:**
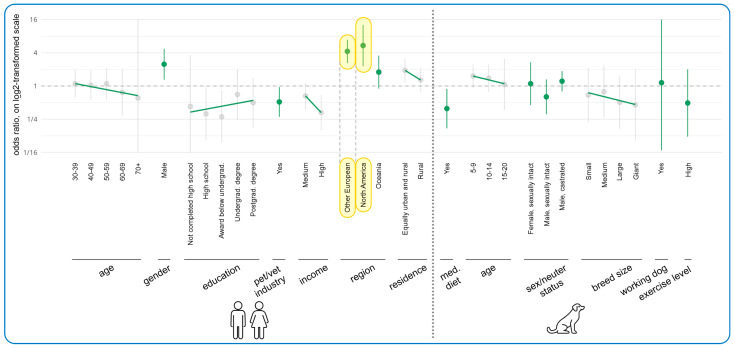
Logistic regression results on the associations between human/dog demographic characteristics and the likelihood of *currently* feeding vegan diets to dogs, relative to the reference characteristics for each group. Note: Vegan dog diets were almost exclusively (91.3%) fed by people who were vegan themselves. As this association was so strong, this regression analysis was run using only vegan respondents. The reference characteristics for these humans and dogs were, respectively: (human) aged 18–29, female, doctoral degree, not in pet/vet industry, low income, UK- and urban-based; (dog) no medical diet, aged 0–4, female, spayed, toy size, a companion dog, and normal exercise levels. Effects are depicted as odds ratios, including 95% confidence intervals (not corrected for multiple testing). Effects that are significant after multiple testing correction are highlighted with yellow bubbles. Individual estimated effects of ordinal variables are grayed out in favor of an additional linear trend line.

Figure 5 displays the results of the regression modeling regarding associations between human and dog demographics and likelihood of *currently* feeding dogs vegan diets. This model only included vegan guardians as of the total 332 guardians feeding a vegan diet, 91.3% (303/332) were vegan themselves. In relation to human demographics, the most apparent tendencies concerned region, gender, working in the pet/vet industries, education level, and income. North Americans had a 441% (Odds Ratio = +441%, 95% Confidence Interval: [+129%, +1180%], *p* = 0.0051), and Other Europeans (i.e., excluding UK) a 324% (OR = +324%, CI: [+161%, +591%], *p* < 0.0001), increased likelihood of feeding their dogs vegan, relative to those in the UK. These both constituted effects. Males likewise had a 148% increased chance of feeding their dogs a vegan diet relative to females; this constituted a trend (OR = +148%, CI: [+30%, +374%], *p* = 0.2313).

In contrast, relative to not working in the pet/vet industries, working in such industries reduced the chance of feeding dogs a vegan diet by 49% (OR = −49%, CI: [−72%, −4%], *p* > 0.9999), and high income by 67% relative to low income (OR = −67%, CI: [−84%, −31%], *p* = 0.1249). These both constituted trends. Relative to having a doctorate, completion of high school and another award below undergraduate degree also reduced the chances of feeding dogs a vegan diet by 69% (OR = −69%, CI: [−90%, −4%], *p* > 0.9999) and 72% (OR = −72%, CI: [−91%, −19%], *p* = 0.6641), respectively. These both constituted trends.

In relation to dog demographics, the only noteworthy tendency was dogs progressing onto a medical diet being 61% less likely to be fed vegan than those not progressing onto medical diets (OR = −61%, CI: [−83%, −11%], *p* = 0.8503). This constituted a trend. See Supplementary Figure S1 for the impact of human and dog demographics on the likelihood of guardians to feed their dogs a raw meat diet.

#### 3.2.2. Current Diet Purchasing Determinants

**Figure 6:**
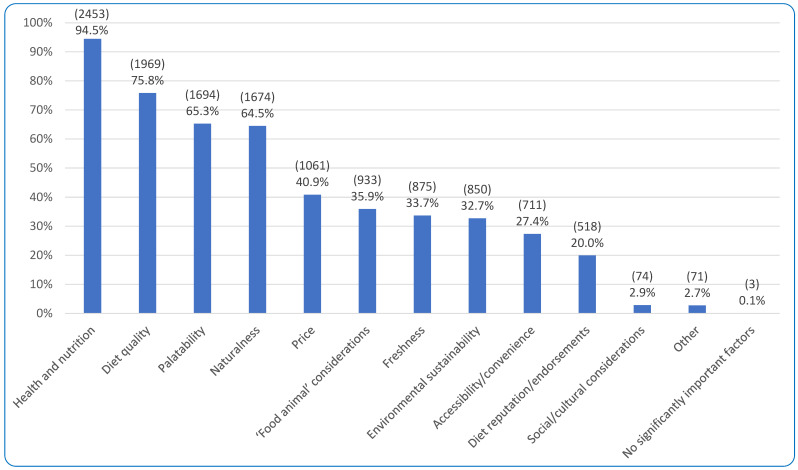
Distribution of current dog food purchasing determinants. Note: Multiple responses were possible; thus, the percentages and counts represent the proportion of all respondents (n = 2596) selecting each answer option. The ‘Other’ responses (2.7%, n = 71) comprised, for instance, ‘WSAVA standards compliant’, whether the food is vegan/organic, and avoiding allergens.

The vast majority of respondents (95.9%, 2489/2596) were the primary decision maker for pet food purchases with 4.1% (107/2596) playing a lesser role. Figure 6 portrays the most commonly selected factors of importance when choosing a dog food, with ‘Health and nutrition’ being the most popular option (selected by 94.5% of respondents, 2453/2596). When asked which health/nutritional factors were specifically important among these 94.5% of respondents, 90.1% (2211/2453) selected ‘Maintenance of pet health,’ 73.3% (1799/2453) selected ‘Nutritional soundness,’ 39.5% (968/2453) ‘Life stage suitability,’ 10.5% (258/2453) ‘Performance on diet,’ and 1.9% (47/2453) ‘Other.’ Examples of ‘Other’ included whether allergens were present, effect on bowels/stools, and safety (no recalls). When asked if there were any particular nutrients these respondents wanted included in pet food, 76.5% (1348/1763) stated ‘No’ and 23.5% (415/1763) stated ‘Yes.’ From the 412 respondents providing qualitative details following their ‘Yes’ response, common responses included all essential nutrients, meeting/exceeding FEDIAF-required levels, B12, L-carnitine, taurine, omega oils, glucosamine, chondroitin, probiotics, prebiotics, and vitamin D.

Of the 20.0% (518/2596) of respondents selecting ‘Diet reputation/endorsements’ as important (Figure 6), 510 answered the question about which endorsements they would like. A good reputation without specific endorsement was selected by 59.8% (305/510) of respondents. ‘Endorsement by veterinarians’ was selected by 37.5% (191/510), ‘Endorsement by others’ by 26.7% (136/510), and ‘Endorsement by other veterinary staff’ by 9.6% (49/510). ‘Endorsement by others’ was elaborated upon by 121 respondents; common answers included the ‘All about dog food’ website, animal behaviorists, animal nutritionists, breed experts, fellow dog guardians (same breed), others experienced with the way respondents’ dogs are fed (e.g., raw, vegan), academic researchers, WAVSA (World Small Animal Veterinary Association).

Of the 35.9% (933/2596) of respondents selecting considerations about ‘food’ animals as an important factor in dog food purchases (Figure 6), 916 clarified further by selecting more specific considerations. ‘The welfare of “food” animals’ was selected by 83.0%, (760/916), ‘The rights of “food” animals’ by 60.4% (553/916), and ‘Other’ by 4.5% (41/916). Of the 2.9% (74/2596) selecting ‘Social or cultural considerations’ as an important factor in pet food purchases, 71 clarified further; the most common concern was ‘Country of origin of ingredients or finished product’ (70.4%, 50/71), followed by ‘Employment of farmers/workers’ (43.7%, 31/71). ‘Social or cultural considerations’ was selected by 32.4% (23/71) and ‘Other’ by 11.3% (8/71).

**Figure 7:**
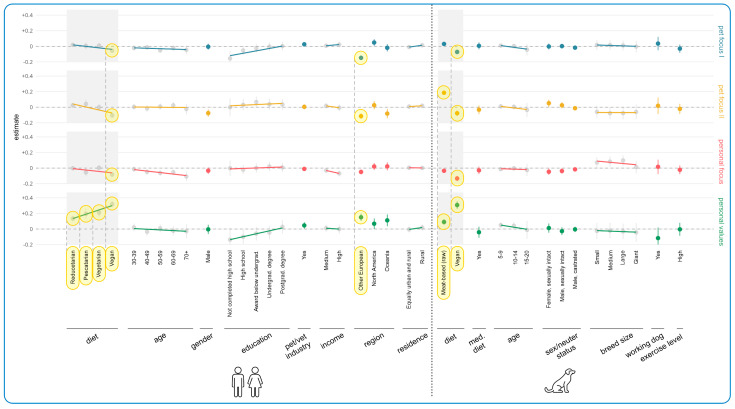
Linear regression results on the associations between human/dog demographic characteristics and current dog food purchasing determinants, relative to the reference characteristics for each group. Note: The reference characteristics for these humans and dogs were, respectively: (human) omnivore, aged 18–29, female, doctoral degree, not in pet/vet industry, low income, UK- and urban-based; (dog) meat-based (conventional), no medical diet, aged 0–4, female, spayed, toy size, a companion dog, and normal exercise levels. The right *y*-axis purchasing determinant categories comprise the following subitems: Pet Focus I (health and nutrition, palatability, diet quality), Pet Focus II (naturalness, freshness, diet reputation), Personal Focus (price, convenience, social/cultural aspects), and Personal Values (concerns about ‘food’ animals or sustainability). Each dependent variable (e.g., Pet Focus I) comprises a score between 0 and 1, reflecting the share of its underlying set of items (see Figures S2 and S3) that respondents ticked in the questionnaire. Effects are depicted including 95% confidence intervals (not corrected for multiple testing). Effects that are significant after multiple testing correction are highlighted with yellow bubbles. Individual estimated effects of ordinal variables are grayed out in favor of an additional linear trend line. Effects of human and dog diet are highlighted through gray-shaded areas to reflect their separate estimation scheme (i.e., these effects were estimated while not controlling for further human demographic variables) as outlined in Section 2.2.

The results of the regression modeling of associations between human and dog demographics and current purchasing determinants are shown in Figure 7. All human demographics other than residence had some impact, especially guardian diet, region, and age. In terms of guardian diet, there was a decreasing tendency of importance of Pet Focus I, Pet Focus II, and Personal Focus with decreasing levels of animal product consumption. This constituted an effect for vegans relative to omnivores in each of these categories (Pet Focus I: Estimate = −0.06, CI: [−0.08, −0.03], *p* = 0.0049); Pet Focus II: Estimate = −0.10, CI: [−0.14, −0.07], *p* < 0.0001); Personal Focus: Estimate = −0.08, CI: [−0.11, −0.05], *p* < 0.0001), and a trend for pescatarians in the Personal Focus category (Estimate = −0.06, CI: [−0.10, −0.01], *p* > 0.9999). Supplementary Figure S2 shows this applied similarly across all items for Pet Focus I and Pet Focus II. But the reverse tendency was evident for the social/cultural subitem of the Personal Focus category, i.e., an *increasing* level of importance as animal products were reduced. This demonstrates that the aforementioned decreasing tendency on the main figure only applied to price and convenience subitems for the Personal Focus category. In contrast, for the Personal Values category, there was a clear increasing tendency in how important this factor became in decisions about pet food with decreasing animal consumption. Relative to omnivores, all dietary categories rated Personal Values as more important, and these all constituted effects (vegan: Estimate = +0.32 (CI: [+0.28, +0.36], *p* < 0.0001); vegetarian: Estimate = +0.21, CI: [+0.16, +0.26], *p* < 0.0001); pescatarian: Estimate = +0.19, CI: [+0.13, +0.26], *p* < 0.0001); reducetarian: Estimate = +0.13, CI: [+0.10, +0.17], *p* < 0.0001). Supplementary Figure S2 shows this was similar across all subitems.

In terms of region, Other Europeans rated Pet Focus I, Pet Focus II, and Personal Focus less importantly than those from the UK. This constituted an effect for the first two aforementioned categories (Estimate = −0.15, CI: [−0.18, −0.12], *p* < 0.0001; Estimate = −0.11, CI: [−0.15, −0.08], *p* < 0.0001), and a trend for the latter (Estimate = −0.05, CI: [−0.08, −0.02], *p* = 0.3384). Trends were also found regarding North Americans rating Pet Focus I more importantly than those from the UK (Estimate = +0.05, CI: [+0.01, +0.09], *p* > 0.9999), and those from Oceania rating Pet Focus II less importantly than those from the UK (Estimate = −0.08, CI: [−0.14, −0.02], *p* > 0.9999). Supplementary Figure S2 shows the reduced importance of Pet Focus I for Other Europeans was mainly in relation to palatability and diet quality (not health). It also shows that the social/cultural subitem of Personal Focus was rated considerably more important than price and convenience by all regions relative to UK. Additionally, all regions rated Personal Values as more important relative to the UK. The share of Personal Value items ticked by Other Europeans was on average 15 percentage points higher than those in the UK, which constituted an effect (Estimate = +0.15, CI: [+0.10, +0.20], *p* < 0.0001). Trends were also found for North Americans (Estimate = +0.07, CI: [+0.001, +0.13], *p* > 0.9999) and those from Oceania (Estimate = +0.11 (CI: [+0.03, +0.19], *p* = 0.7455) relative to the UK.

With regard to age, there was a decreasing tendency to rate the Personal Focus category as important with increasing age. Relative to the 18–29 age group, four trends were found regarding this (ages 40–49: estimate = −0.05, CI: [−0.08, −0.01], *p* > 0.9999; ages 50–59: estimate = −0.06, CI: [−0.10, −0.03], *p* = 0.0930; ages 60–69: estimate = −0.05, CI: [−0.09, −0.02], *p* > 0.9999; and ages 70+: estimate = −0.11, CI: [−0.17, −0.05], *p* = 0.0608). There was also a tendency to find Pet Focus I and Personal Vaues increasingly more important with increasing education. Regarding the Pet Focus I category, one trend was found for those not completing high school relative to those with a doctorate (Estimate = −0.16, CI: [−0.25, −0.06], *p* = 0.1927). Regarding the Personal Values category, a further trend was found for this same tendency for guardians completing high school relative to those with a doctorate (Estimate = −0.10, CI: [−0.19, −0.01], *p* > 0.9999). Finally, regarding income, Personal Focus received lower ratings of importance with increasing income. There was a trend for both medium (Estimate = −0.03, CI: [−0.06, −0.0001], *p* > 0.9999) and high (Estimate = −0.07, CI: [−0.11, −0.03], *p* > 0.9999), relative to low income. Specifically, Supplementary Figure S2 shows a 39% reduced chance of the price subitem being ticked by guardians on a high income compared to those on a low income (OR = −39%, CI: [−52%, −23%]). This constituted an explorative tendency (Table S1b).

In terms of dog demographics, the main variable impacting on current pet food purchasing determinants was dog diet. Compared to guardians feeding their dogs a conventional meat-based diet, guardians feeding a vegan diet rated all categories (except Personal Values) significantly less importantly (Pet Focus I: estimate = −0.07, CI: [−0.10, −0.04], *p* = 0.0008); Pet Focus II: estimate = −0.08, CI: [−0.11, −0.04], *p* = 0.0047); Personal Focus: estimate = −0.13, CI: [−0.16, −0.10], *p* < 0.0001). These constituted three effects. The converse was true for Personal Values; those feeding a vegan diet rated this category significantly more important than those feeding a conventional meat-based diet (Estimate = +0.31, CI: [+0.26, +0.35], *p* < 0.0001). This likewise constituted an effect.

Similarly to those feeding a vegan diet, those feeding a raw meat diet also rated the Personal Values category significantly more important (Estimate = +0.09, CI: [+0.06, +0.12], *p* < 0.0001), and the Personal Focus category significantly less important (Estimate = −0.03, CI: [−0.06, −0.01], *p* = 0.6322), relative to those feeding a conventional meat-based diet. The former constituted an effect and the latter a trend. However, in contrast to those feeding a vegan diet, those feeding a raw meat diet rated the Pet Focus I (Estimate = +0.03, CI: [+0.01, +0.05], *p* > 0.9999) and Pet Focus II (Estimate = +0.19, CI: [+0.16, +0.21], *p* < 0.0001) categories more important than those feeding a conventional meat-based diet. These constituted a trend and an effect, respectively. Additionally, Supplementary Figure S3 shows this was in relation to the subitems naturalness and freshness (vs. reputation) for Pet Focus II. This figure also shows that the health/nutrition subitem of Pet Focus I was not applicable, nor the social/cultural subitem of Personal Focus. All other subitems scored similarly.

### 3.3. Acceptance and Essential Characteristics of More Sustainable Dog Diets

#### 3.3.1. Acceptance of More Sustainable Dog Diets

Those feeding conventional or raw meat-based diets (collectively 84.2%, 2188/2596) were asked which alternative food types they might realistically consider, assuming all their desired attributes could be met—2169 responded. Table 2 demonstrates how all of the alternatives remained *un*acceptable to 56.8% (1231/2169). Cultivated meat-based food was the next most common selection, chosen by 24.4% (529/2169) of respondents. Vegetarian, insect-based, and vegan options were selected by respondents to comparable extents (16.6%, 359/2169; 15.5%, 336/2169; and 13.4%, 290/2169, respectively). Included in these figures are 10 respondents who deemed all options unacceptable, but who also simultaneously selected one of the alternatives as acceptable (cultivated meat, n = 4; vegetarian, n = 3; vegan, n = 1; insect-based, n = 2).

**Figure 8:**
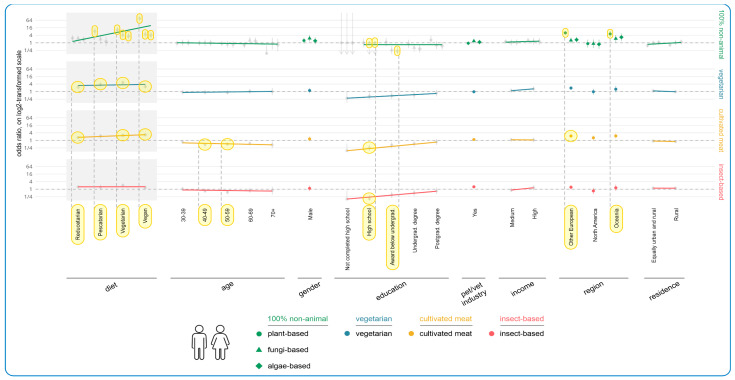
Logistic regression results on the associations between human demographic characteristics and the acceptance of more sustainable dog diets, among guardians currently feeding meat-based dog food (raw or conventional), relative to the reference characteristics for each group. Note: The reference characteristics for these people were: omnivore, aged 18–29, female, doctoral degree, not in pet/vet industry, low income, UK- and urban-based. Effects are depicted as odds ratios, including 95% confidence intervals (not corrected for multiple testing). Effects that are significant after multiple testing correction are highlighted with yellow bubbles. Individual estimated effects of ordinal variables are grayed out in favor of an additional linear trend line. Effects of human diet are highlighted through gray-shaded areas to reflect their separate estimation scheme (i.e., these effects were estimated while not controlling for further human demographic variables) as outlined in Section 2.2.

**Figure 9:**
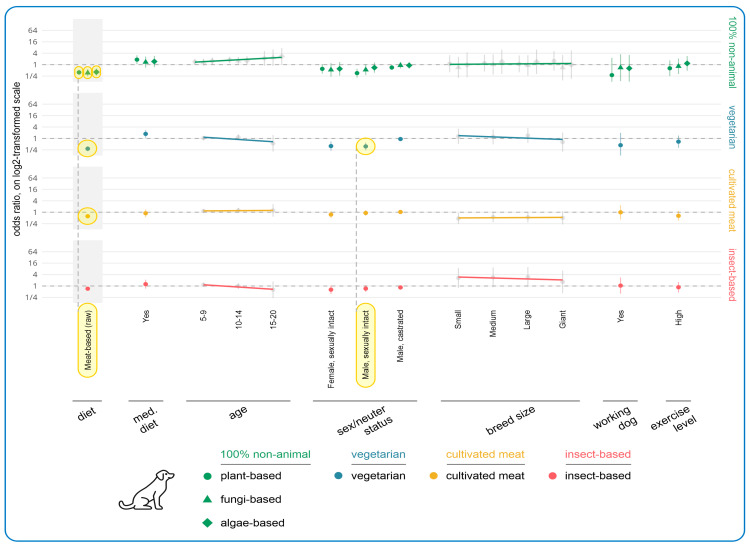
Logistic regression results on the associations between dog demographic characteristics and the acceptance of sustainable dog diets, relative to the reference characteristics for each group. Note: The reference characteristics for these dogs were: meat-based (conventional), no medical diet, aged 0–4, female, spayed, toy size, a companion dog, and normal exercise levels. Effects are depicted as odds ratios, including 95% confidence intervals (not corrected for multiple testing). Effects that are significant after multiple testing correction are highlighted with yellow bubbles. Individual estimated effects of ordinal variables are grayed out in favor of an additional linear trend line. Effects of dog diet are highlighted through gray-shaded areas to reflect their separate estimation scheme (i.e., these effects were estimated while not controlling for further human demographic variables) as outlined in Section 2.2.

The results of the regression modeling of associations between human and dog demographics and acceptance of alternative more sustainable dog diets are shown in Figure 8 and Figure 9, respectively. The main tendencies found among human demographic variables concerned guardian diet, age, education, and region. In terms of guardian diet, there were clear tendencies of greater acceptance of all alternative diets (bar insect-based food). Vegans and vegetarians were significantly more likely to accept all 100% animal-free options, relative to omnivores. This resulted in six effects, as follows: plant-based (vegan: OR = +8662%, CI: [+5216%, +14,341%], *p* < 0.0001; vegetarian: OR = +972%, CI: [+538%, +1702%], *p* < 0.0001); fungi-based (vegan: OR = +375%, CI: [+188%, +684%], *p* < 0.0001; vegetarian: OR = +322%, CI: [+152%, +605%], *p* < 0.0001); and algae-based (vegan: OR = +304%, CI: [+145%, +565%], *p* < 0.0001; vegetarian: OR = +241%, CI: [+103%, +472%], *p* = 0.0011). An effect was also found regarding pescatarians’ higher acceptance of plant-based alternatives relative to omnivores (OR = +736%, CI: [+346%, +1468%], *p* < 0.0001).

Relative to omnivore guardians, four effects were also found regarding higher acceptance of vegetarian diets among reducetarians (OR = +123%, CI: [+63%, +205%], *p* = 0.0001), pescatarians (OR = +266%, CI: [+131%, +481%], *p* < 0.0001), vegetarians (OR = +424%, CI: [+268%, +646%], *p* < 0.0001), and vegans (OR = +149%, CI: [+68%, +267%], *p* = 0.0015). For vegans, there was a slight dip regarding the general tendency of increased acceptance of vegetarian diets with reduced animal consumption. For cultivated meat, three effects were found regarding reducetarians’ (OR = +70%, CI: [+32%, +120%], *p* = 0.0119), vegetarians’ (OR = +152%, CI: [+83%, +246%], *p* < 0.0001), and vegans’ (OR = +184%, CI: [+107%, +289%], *p* < 0.0001) acceptance of this alternative relative to omnivores, and one trend for pescatarians (OR = +119%, CI: [+44%, +232%], *p* = 0.0749).

In terms of age, there was a slight decreasing acceptance of cultivated meat with increasing guardian age. Two effects were found for the 40–49 (OR = −55%, CI: [−68%, −36%], *p* = 0.0020) and 50–59 (OR = −50%, CI: [−64%, −30%], *p* = 0.0183) age groups, relative to ages 18–29. The acceptability of plant-based alternatives was also 86% lower for guardians aged 70+, compared relative to ages 18–29. This constituted a trend (OR = −86%, CI: [−97%, −38%], *p* > 0.9999).

In terms of education, there were clear tendencies of higher acceptance of alternative diets (except 100% animal-free) with increasing education levels. For cultivated meat, there was one effect; namely, those with high school as the highest education level accepted cultivated meat significantly less than guardians with a doctorate (OR = −75%, CI: [−86%, −55%], *p* = 0.0016). One effect was found regarding lower acceptance of insect-based alternatives among those completing high school relative to guardians with a doctorate (OR = −83%, CI: [−91%, −65%], *p* = 0.0003). Eight additional trends were also found in support of the tendency towards higher acceptance of more sustainable alternative dog diets with increasing education.

With regard to 100% animal-free diets, three education effects were found; namely, those completing high school were significantly less likely to accept fungi-based (OR = −88%, CI: [−95%, −70%], *p* = 0.0025) and algae-based (OR = −90%, CI: [−96%, −75%], *p* = 0.0003) dog diets, and those with an award below undergraduate degree level were significantly less likely to accept algae-based alternatives (OR = −78%, CI: [−90%, −54%], *p* = 0.0221), relative to guardians with a doctorate. Three trends were also found.

The region guardians lived in showed three further effects. Plant-based diets were considered significantly more acceptable by Other Europeans (OR = +522%, CI: [+343%, +791%], *p* < 0.0001) and guardians from Oceania (OR = +417%, CI: [+198%, +797%], *p* < 0.0001), relative to those from the UK, while Other Europeans were also significantly more likely to accept cultivated meat-based dog food (OR = +124%, CI: [+61%, +211%], *p* = 0.0004), relative to those from the UK. These constituted three effects.

In terms of dog demographics, among the guardians currently feeding conventional or raw meat-based dog food, the key variables impacting acceptance of alternatives were current dog diet, dog age, and sex/neuter status. Guardians currently feeding raw meat were significantly less accepting of virtually all alternatives, relative to those feeding a conventional meat-based diet. This generated five effects: plant-based (OR = −61%, CI: [−71%, −47%], *p* < 0.0001); fungi-based (OR = −63%, CI: [−76%, −43%], *p* = 0.0014); algae-based (OR = −59%, CI: [−73%, −38%], p = 0.0105); vegetarian (OR = −71%, CI: [−79%, −61%], *p* < 0.0001); and cultivated meat (OR = −39%, CI: [−50%, −24%], *p* = 0.0029).

One effect was also found regarding guardians of male sexually intact dogs being significantly less likely to accept vegetarian dog diets (OR = −62%, CI: [−76%, −40%], *p* = 0.0130). This reflected a general tendency for guardians of both female and male sexually intact dogs to be less accepting of more sustainable alternatives, for which there were two trends. In terms of dog age, there was a clear increasing tendency to accept animal-free foods as the age of dogs increased. For instance, the chance that plant-based dog food was considered acceptable was 42% higher for guardians whose dog was 5–9 years old (OR = +42%, CI: [+3%, +97%], *p* > 0.9999), and 77% higher for guardians whose dog was 10–14 years (OR = +77%, CI: [+19%, +164%], *p* > 0.9999), compared to guardians of dogs aged 0–4 years. These both constituted trends. Additionally, guardians of dogs progressing onto a medical diet were more accepting of plant-based dog diets (OR = +84%, CI: [+8%, +212%], *p* > 0.9999) and vegetarian dog diets (OR = +73%, CI: [+8%, +177%], *p* > 0.9999), relative to guardians of dogs not progressing onto a medical diet. These also constituted trends.

#### 3.3.2. Essential Characteristics of Alternative Dog Diets

**Figure 10:**
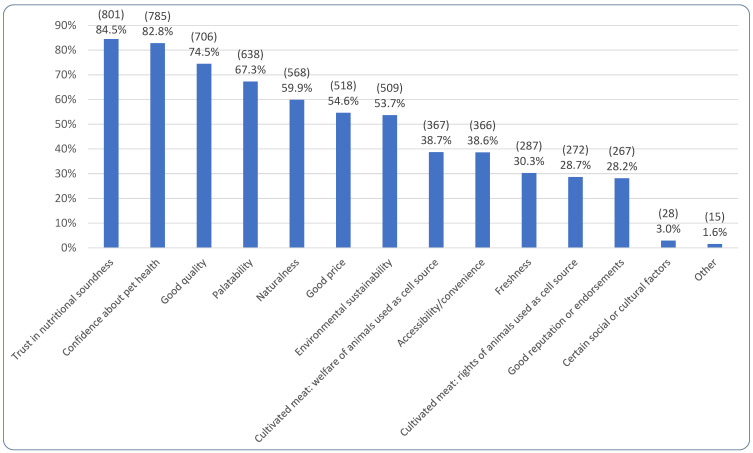
Distribution of attributes of sustainable dog food alternatives considered essential by guardians currently feeding conventional/raw meat-based dog food who stated they may realistically consider a sustainable alternative. Note: These attributes were considered essential in order for these respondents to realistically consider choosing alternative options. Multiple responses were possible; thus, the percentages and counts represent the proportion of all respondents (n = 948) selecting each answer option. Among these respondents are 10 respondents who deemed all options unacceptable, but who also simultaneously selected one of the alternatives as acceptable (cultivated meat, n = 4; vegetarian, n = 3; vegan, n = 1; insect-based, n = 2). Common ‘Other’ answers (1.6%, n = 15) included allergen-free, evidence-based nutrition including bioavailability, specific health concerns such as skin problems and gastrointestinal reactions, and insect welfare.

Figure 10 portrays the frequencies with which different attributes were considered essential in order for alternative dog foods to be chosen, by guardians open to more sustainable alternative dog diets. Confidence about nutritional soundness was selected most commonly (by 84.5%, 801/948). When these respondents were asked if there were specific nutrients alternative foods would need to provide, 80.8% (647/801) said ‘No’ with the remainder stating ‘Yes’ (19.2%, 154/801). Those selecting ‘Yes,’ were asked which nutrients would be necessary; among 154 responses, common answers included ‘all essential nutrients,’ exceeding FEDIAF/AAFCO/NRC/WSAVA minimum requirements, B12, vitamin D, omegas, glucosamine, chondroitin, ‘the same amino acid profile as meat,’ prebiotics, probiotics, protein, and taurine.

Of the 28.2% (267/948) selecting a ‘Good reputation or endorsements’ as essential, 51.3% (137/267) selected ‘A good reputation without specific endorsements would be enough,’ 46.4% (124/267) selected ‘Endorsements by veterinarians,’ 21.0% (56/267) selected ‘Endorsement by others,’ and 12.4% (33/267) selected ‘Endorsement by other veterinary staff.’ Of the 21.0% (56/267) selecting ‘Endorsement by others,’ common ‘Other’ responses included ‘Canine nutritionist’, the ‘All about dog food’ website, ‘Canine behaviorists’, animal welfare organizations, dog guardians, breeders, and independent researchers. Among the 3.0% (28/948) selecting ‘Certain social or cultural factors,’ 82.1% (23/28) selected ‘Country of origin of ingredients or finished product’ as important, 53.6% (15/28) selected ‘Employment of farmers and workers,’ 21.4% (6/28) selected ‘Cultural or religious factors,’ and 10.7% (3/28) selected ‘Other’ (e.g., ‘humanely reared’ and ‘not Halal’).

**Figure 11:**
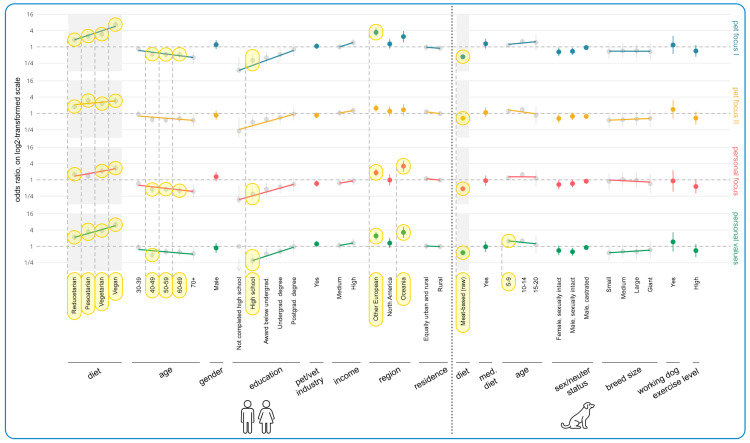
Logistic regression results on the associations between human/dog demographic characteristics and characteristics of more sustainable dog diets considered essential, among guardians currently feeding meat-based dog food (raw or conventional), relative to the reference characteristics for each group. Note: The reference characteristics for these humans and dogs were, respectively, as follows: human: omnivore, aged 18–29, female, doctoral degree, not in pet/vet industry, low income, UK- and urban-based; dog: meat-based (conventional), no medical diet, aged 0–4, female, spayed, toy size, a companion dog, and normal exercise levels. The right *y*-axis essential characteristic categories comprise the following subitems: Pet Focus I (health, nutrition, palatability, diet quality), Pet Focus II (naturalness, freshness, diet reputation), Personal Focus (price, convenience, social/cultural aspects), and Personal Values (sustainability and animal welfare/rights regarding source animals used for cultivated meat-based dog food). Each dependent variable (e.g., Pet Focus I) reflects the information if at least one item among its underlying set of items (see Figures S4 and S5) was ticked in the questionnaire. Effects are depicted as odds ratios, including 95% confidence intervals (not corrected for multiple testing). Effects that are significant after multiple testing correction are highlighted with yellow bubbles. Individual estimated effects of ordinal variables are grayed out in favor of an additional linear trend line. Effects of human and dog diet are highlighted through gray-shaded areas to reflect their separate estimation scheme (i.e., these effects were estimated while not controlling for further human demographic variables) as outlined in Section 2.2.

Figure 11 shows the results of the regression modeling of associations between human and dog demographics and essential characteristics that alternative diets would require for them to realistically be chosen. In terms of human demographics, the most noteworthy results concerned guardian diet, age, education, and region. There was a clear tendency for all essential characteristic categories to increase in importance with reduced animal consumption. This resulted in effects for *all* dietary types and categories (15 in total), except pescatarian and Personal Focus (which was also not a trend). To take just one pair of examples (of the 15 in total) from opposite ends of the dietary spectrum, vegans were 572% more likely to find Pet Focus I important (OR = +572%, CI: [+395%, +813%], *p* < 0.0001), relative to omnivores, while reducetarians were 82% more likely (OR = +82%, CI: [+46%, +127%], *p* < 0.0001). Supplementary Figure S4 demonstrates this pattern applied across category subitems.

There was also a clear tendency of decreasing importance of all essential characteristic categories with increasing guardian age. This resulted in three effects for the Pet Focus I category: 40–49 (OR = −47%, CI: [−61%, −27%], *p* = 0.0139), 50–59 (OR = −48%, CI: [−61%, −29%], *p* = 0.0059), and 60–69 (OR = −47%, CI: [−62%, −26%], *p* = 0.0326). There were also three effects for the Personal Focus category: 40–49 (OR = −55%, CI: [−68%, −38%], *p* = 0.0003), 50–59 (OR = −55%, CI: [−67%, −37%], *p* = 0.0003), and 60–69 (OR = −60%, CI: [−72%, −42%], *p* = 0.0002). Additionally, there was an effect regarding the tendency for the 40–49 guardian age group to rate Personal Values as more essential (OR = −52%, CI: [−66%, −32%], *p* = 0.0043), relative to those aged 18–29. There were also 10 trends in further support of this tendency (see Supplementary Table S2b).

In terms of education, there were clear tendencies of increasing importance of all essential characteristic categories with increasing education levels. This included three effects—all for guardians who had completed high school and their lower valuing of Pet Focus I (OR = −68%, CI: [−82%, −44%], *p* = 0.0114), Personal Focus (OR = −70%, CI: [−83%, −47%], *p* = 0.0061), and Personal Values (OR = −70%, CI: [−83%, −47%], *p* = 0.0076), relative to guardians with a doctorate. There were also 10 trends in further support of this tendency (see Supplementary Table S2b).

In terms of region, Other Europeans and those from Oceania tended to value all categories more than guardians from the UK, and five further effects were found in this regard: Pet Focus I (Other European: OR = +245%, CI: [+149%, +380%], *p* < 0.0001), Personal Focus (Other European: OR = +84%, CI: [+35%, +152%], *p* = 0.0182), Oceania: OR = +220%, CI: [+99%, +416%], *p* = 0.0003), and Personal Values (Other European: OR = +143%, CI: [+78%, +232%], *p* < 0.0001), Oceania: OR = +228%, CI: [+102%, +431%], *p* = 0.0003). Trends were also found for Pet Focus II regarding Other European (OR = +56%, CI: [+14%, +112%], *p* = 0.7027), and Pet Focus I regarding Oceania (OR = +140%, CI: [+47%, +291%], *p* = 0.0685). Supplementary Figure S4 demonstrates the category subitems were rated fairly similarly for all of the cases above, apart from for a lower rating by Other Europeans for nutritional soundness and palatability in Pet Focus I.

In terms of dog demographics, the key impacting variable was current dog diet. Among the guardians *not* currently feeding a more sustainable alternative, those currently feeding a raw meat-based diet to their dogs were significantly less likely to find all categories important, relative to guardians feeding a conventional diet. This constituted four effects: Pet Focus I (OR = −57%, CI: [−64%, −48%], *p* < 0.0001), Pet Focus II (OR = −34%, CI: [−46%, −19%], *p* = 0.0094), Personal Focus (OR = −53%, CI: [−62%, −42%], *p* < 0.0001), and Personal Values (OR = −42%, CI: [−52%, −28%], *p* < 0.0001). Supplementary Figure S5 shows similar ratings across all subitems except for the freshness subitem in Pet Focus II; it is rated of similar importance as those feeding conventional meat. Additionally, guardians of sexually intact male and female dogs rated all categories less important than guardians of spayed females. For instance, guardians with a female sexually intact dog had a 35% lower chance of having ticked at least one item in the Pet Focus II (OR = −35%, CI: [−56%, −4%], *p* > 0.9999), compared to people with a female spayed dog, and guardians with a male sexually intact dog had a 31% lower chance of having done so for Pet Focus I (OR = −31%, CI: [−49%, −7%], *p* > 0.9999). These both constituted trends. Supplementary Figure S5 shows similar scoring across all subitems, though the reputation subitem within Pet Focus II was especially low.

### 3.4. Dog Diet Information Sources

**Figure 12:**
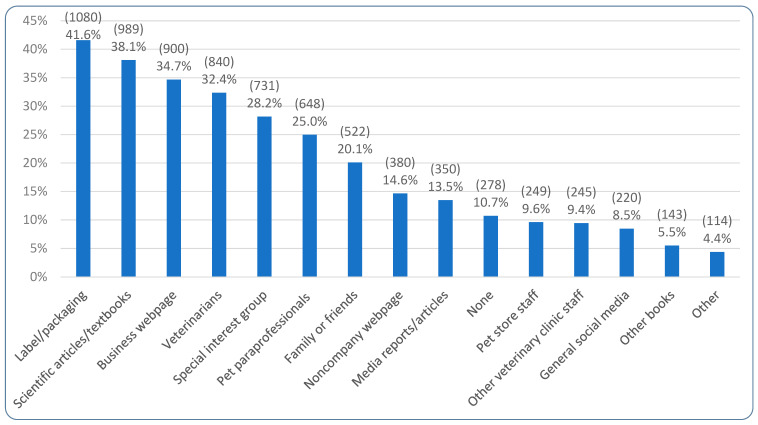
Distribution of information sources significantly influencing respondents’ dog diet choices. Note: Multiple responses were possible; thus, the percentages and counts represent the proportion of all respondents (n = 2596) selecting each answer option. Elaborations on ‘Other’ (4.4%, n = 114) included previous users of a dog food product, animal nutritionists, product awards, adoption charity practice, and ingredients listed (as opposed to other label/packaging details).

Figure 12 demonstrates the information sources that respondents selected as significantly influencing their pet food choices. ‘Label/packaging’ was most commonly selected, by 41.6% (1080/2596). Additionally, 82.9% (2153/2596) reported not having received any nutritional recommendations from veterinary clinic staff in the last year, with 15.0% (389/2596) reporting that they had and 2.1% (54/2596) stating they were unsure. Of those reporting they had, 384 left comments regarding what particular advice they had received. Table 5 summarizes this advice, with a recommendation of ailment-specific food (n = 53), particular brands (n = 47), and supplements (n = 46) being the three most common. Of note is that compliance with the veterinary advice reported by 379 (of 389) was described as ‘Good’ by 60.2% (228/379), ‘Poor’ by 26.4% (100/379), and ‘Medium’ by 13.5% (51/379).

**Figure 13:**
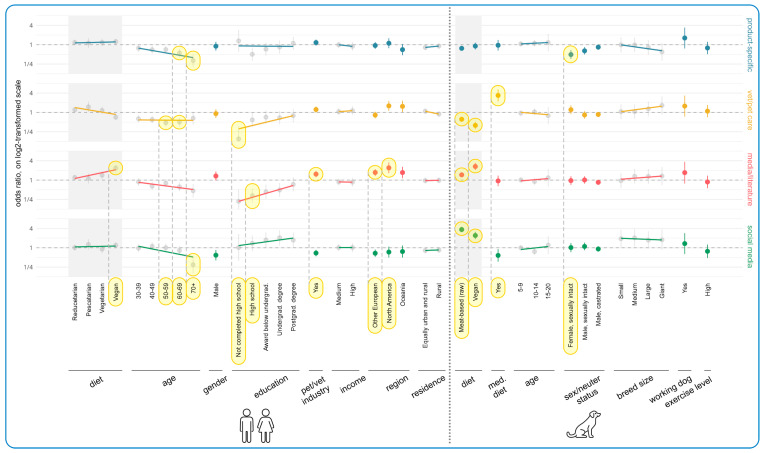
Logistic regression results on the associations between human/dog demographic characteristics and dietary information sources, relative to the reference characteristics for each group. Note: The reference characteristics for these humans and dogs were, respectively, as follows: (human) omnivore, aged 18–29, female, doctoral degree, not in pet/vet industry, low income, UK- and urban-based; (dog) meat-based (conventional), no medical diet, aged 0–4, female, spayed, toy size, a companion dog, and normal exercise levels. The right *y*-axis information source categories comprise the following subitems: Product-Specific (label/packaging, company webpage), Vet/Pet Care (veterinarians, other vet clinic staff, pet store staff, pet paraprofessionals), Media/Literature (scientific literature, media reports, non-company webpages, other books), and Social Media (online special interest groups, general social media). Each dependent variable (e.g., Product-Specific) reflects the information if at least one item among its underlying set of items (see Figures S6 and S7) was ticked in the questionnaire. Effects are depicted as odds ratios, including 95% confidence intervals (not corrected for multiple testing). Effects that are significant after multiple testing correction are highlighted with yellow bubbles. Individual estimated effects of ordinal variables are grayed out in favor of an additional linear trend line. Effects of human and dog diet are highlighted through gray-shaded areas to reflect their separate estimation scheme (i.e., these effects were estimated while not controlling for further human demographic variables) as outlined in Section 2.2.

Figure 13 demonstrates the results of the regression modeling regarding associations between human and dog demographics and information sources used to inform dog diet decisions. The most noteworthy tendencies regarding human demographics concerned guardian diet, age, education level, working in the pet/vet industries, and region. There was a slight increasing tendency to value all categories of information sources with decreasing animal consumption (except Vet/Pet Care). This was particularly the case for Media/Literature with the differences in valuing this category constituting an effect for vegan guardians relative to omnivores; vegans were 137% more likely to use this information source than omnivores (OR = +137%, CI: [+91%, +194%], *p* < 0.0001). Vegetarians were 42% more likely to use Media/Literature relative to omnivores (OR = +42%, CI: [+8%, +87%], *p* > 0.9999), which constituted a trend. A trend was also found in the Product-Specific category insofar as vegans were 25% more likely to use this category relative to omnivores (OR = +25%, CI: [+1%, +54%], *p* > 0.9999). In contrast, while reducetarians, pescatarians, and vegetarians valued Vet/Pet Care to a similar extent to (or slightly more than) omnivores, vegans used this category significantly less than omnivores (OR = −29%, CI: [−43%, −12%], *p* = 0.2604); this constituted a trend. Supplementary Figure S6 shows this was particularly the case for pet paraprofessionals.

In terms of the impact of age, Vet/Pet Care was used less by all age groups than 18–29 year olds. For instance, 50–59 year olds used Vet/Pet Care 52% less than 18–29 year olds (OR = −52%, CI: [−64%, −37%], *p* < 0.0001), and 60–69 year olds 50% less (OR = −50%, CI: [−63%, −32%], *p* = 0.0023). These both constituted effects. There were also two trends for the 30–39 (OR = −37%, CI: [−52%, −17%], *p* = 0.1785) and 40–49 (OR = −40%, CI: [−54%, −21%], *p* = 0.0560) age groups. Supplementary Figure S6 shows that low Vet/Pet Care use did *not* apply to pet paraprofessionals. For the other three information categories, there were clear decreasing tendencies of use as guardian age increased. There were two effects for the 70+ age group regarding their use of Product-Specific sources (OR = −68%, CI: [−80%, −48%], *p* = 0.0005) and Social Media (OR = −70%, CI: [−84%, −45%], *p* = 0.0230); Supplementary Figure S6 shows that low Social Media use primarily concerned the subitem general social media use. There was also one effect for the 60–69 age group in relation to the Product-Specific category (OR = −45%, CI: [−60%, −25%], *p* = 0.0260). There were two trends for the 40–49 (OR = −30%, CI: [−47%, −7%], *p* > 0.9999) and 50–59 (OR = −29%, CI: [−46%, −6%], *p* > 0.9999) age groups in terms of the Product-Specific category as well as three trends for the 40–49 (OR = −35%, CI: [−51%, −14%], *p* = 0.4007), 60–69 (OR = −39%, CI: [−55%, −17%], *p* = 0.3333), and 70+ (OR = −54%, CI: [−71%, −25%], *p* = 0.2971) age groups in terms of the Media/Literature category.

In terms of education, there were clear tendencies of increased information source category use as education level increased, apart from for the Product-Specific category. This included two effects for not completing high school and Vet/Pet Care use (OR = −85%, CI: [−94%, −63%], *p* = 0.0095) and high school as the highest education level achieved and Media/Literature use (OR = −67%, CI: [−80%, −45%], *p* = 0.0041), relative to those with a doctorate. There were also four trends: completing high school and Vet/Pet Care use (OR = −42%, CI: [−64%, −5%], *p* > 0.9999); and not completing high school (OR = −78%, CI: [−91%, −49%], *p* = 0.0805), an award below undergraduate degree (OR = −57%, CI: [−74%, −28%], *p* = 0.1975), and undergraduate degree (OR = −51%, CI: [−70%, −19%], *p* = 0.8701) in terms of Media/Literature use, relative to those with a doctorate. Supplementary Figure S6 shows pet store staff are used more than other subitems in Vet/Pet Care, media reports more than other subitems in Media/Literature, and that the subitems are all rated quite similarly for Social Media.

Those working in the pet/vet industries were 53% *more* likely than those not working in such industries to use Media/Literature and 31% *less* likely to use Social Media for pet food decisions. This constituted an effect (OR = +53%, CI: [+23%, +91%], *p* = 0.0242) and a trend (OR = −31%, CI: [−46%, −13%], *p* = 0.2604), respectively. Supplementary Figure S6 shows the Media/Literature use concerned scientific literature and other books. Additionally, all other regions (Other European, North American, and Oceania) all used Media/Literature more than those in the UK. This constituted two effects (OR = +72%, CI: [+35%, +120%], *p* = 0.0030; OR = +141%, CI: [+64%, +254%], *p* = 0.0016) and a trend (OR = +72%, CI: [+14%, +159%], *p* > 0.9999), respectively. Supplementary Figure S6 shows this particularly related to the subitem scientific literature use. There were no effects found in relation to gender, though males were 41% less likely to use Social Media (OR = −41%, CI: [−59%, −15%], *p* = 0.6890) than females, which was a trend; Supplementary Figure S6 shows this was similar across subitems.

The most noteworthy tendencies regarding dog demographics concerned dog diet, medical diet, sex/neuter status, and breed size. In terms of dog diet, guardians feeding both raw meat and vegan diets used Product-Specific and Vet/Pet Care categories less than those feeding conventional meat. This resulted in two effects in the Vet/Pet Care category (vegan: OR = −61%, CI: [−69%, −49%], *p* < 0.0001; raw meat: OR = −39%, CI: [−49%, −27%], *p* < 0.0001), and a trend in the Product-Specific category (raw meat: OR = −23%, CI: [−35%, −8%], *p* = 0.7351). Supplementary Figure S7 shows that for those feeding raw meat the low rating particularly applied to veterinarians; for those feeding vegan, veterinarians were the only subitem *not* rated low relative to those feeding a conventional meat diet. In contrast, guardians feeding both raw meat and vegan diets had significantly increased use of Media/literature and Social Media, relative to guardians feeding a conventional meat diet. This resulted in two effects for Media/Literature (vegan: OR = +165%, CI: [+104%, +243%], *p* < 0.0001; raw meat: OR = +46%, CI: [+22%, +74%], *p* = 0.0066), and two effects for Social Media (vegan: OR = +141%, CI: [+86%, +212%], *p* < 0.0001); raw meat: OR = +269%, CI: [+204%, +347%], *p* < 0.0001). Supplementary Figure S7 shows the subitems ‘scientific literature’ and ‘other books’ were particularly applicable to Media/Literature (although scientific literature was lower for raw meat feeders) and that special interest groups were particularly applicable to Social Media, especially for those feeding raw meat.

Guardians of dogs who progressed onto a medical diet were 237% more likely to use Vet/Pet Care as an information source relative to guardians of dogs not progressing onto a medical diet (OR = +237%, CI: [+118%, +419%], *p* < 0.0001); this constituted an effect. Supplementary Figure S7 shows this pertained to veterinarians and other veterinary staff only. There were tendencies regarding guardians of sexually intact dogs using the Product-Specific category less than guardians of female, spayed dogs. This constituted one effect insofar as guardians of female, sexually intact dogs were 51% less likely to use this category (OR = −51%, CI: [−64%, −32%], *p* = 0.0021), and one trend insofar as guardians of male, sexually intact dogs were 35% less likely to use this category (OR = −35%, CI: [−50%, −16%], *p* = 0.2035). Supplementary Figure S7 shows this applied across all subitems. No other impact of sex/neuter status was found.

## 4. Discussion

This study aimed to gain insights into (1) dog guardians’ current feeding patterns and dog food purchasing determinants, (2) acceptance of more sustainable dog diets, and (3) sources of information used for decisions about dog diets. This section discusses the most important results regarding each of these three main parts of this research. Current feeding practices and purchasing determinants are first discussed, followed by acceptance of alternative sustainable dog food options, and finally, dog food information sources used by guardians. The section finishes with consideration of this study’s limitations, and with recommendations for stakeholders within the dog food industry. The discussion centers primarily on feeding vegan diets to dogs as this is the primary focus of the study as explained in Section 1.2.

### 4.1. Current Feeding Practices and Purchasing Determinants

The present study found that 52% of dog guardians fed a primarily conventional meat-based diet to their dogs and that over 71% of guardians’ dogs diets comprised 75% or more commercial dog food. It also found the top three means of acquiring dog food were direct from the manufacturer, online, or from a pet store. Direct comparisons with findings from other studies are difficult due to varying wording or question types in different surveys. Nevertheless, broadly speaking, this finding is reflected elsewhere in the literature. For instance, in an international survey of dog and cat guardians, Dodd et al. [46] found that 78% of dog guardians at least partially fed their dogs a conventional (commercial, heat-processed) diet, with 79% of these guardians doing so daily. This amounts to 63% of the overall total number of dog guardians surveyed. While this figure is 11% higher than our corresponding figure, the question wording was different to that of the present study. It enquired about *frequency* of feeding (with *daily* being the highest frequency). Daily feeding does not *necessarily* equate to the *primary* food provided, which is what was enquired about in the present survey. Hence, one could expect the 63% figure to reduce a little on account of these differences. Additionally, Dodd et al. [46] did not differentiate between vegan and nonvegan conventional dog food for this specific survey question, which may also reduce the 63% figure slightly if only meat-based conventional dog food is considered.

In a UK-based survey of dog and cat guardians, Hunter and Murison [60] found just under 60% of dog guardians fed wet, dry, or both wet and dry commercial dog food and that online purchases direct from the manufacturer were the most common means of acquiring pet food. However, they did not distinguish between dogs and cats. Dodd et al. [46] also found the top three means of acquiring pet food to be pet stores, supermarkets, or through online distributors. Again, they did not differentiate between dog and cat guardians. The lack of differentiation between dog and cat guardians may account for the slight differences in results when compared to the present study. Higher figures for feeding primarily commercial kibble or canned food have been found in two further surveys: 86% [44] and almost 80% [45]. In both cases, the surveys were limited to the USA (implicitly, in the study by Schleicher et al. [45]) and guardians feeding vegan commercial pet food were not differentiated. In the latter case [45], dogs and cats were not differentiated between. In contrast to the aforementioned studies by Dodd et al. [46] and Hunter and Murison [60], Schleicher et al. [45] found that large pet stores, “Other,” or small pet stores were the three most popular means of purchasing pet food. Different terminology, combining dog/cat guardians, and different survey answer options may be partially responsible for the variations in top answers. Indeed, acquisition through online means was not listed as an option in the study by Schleicher et al. [45] and ‘Other’ was the second highest option at over 22%. Respondents purchasing pet food online may have selected “Other,” in this case, which would be congruent with the results from aforementioned studies.

The alternative diets making up the dog diet choices of the remaining 48% of guardians comprised primarily raw meat (32%) and vegan diets (13%). The present study found a substantially higher percentage of dog guardians feeding a raw meat-based diet, relative to the 3%, 9%, and 19% in studies by APPA [61], Dodd et al. [46], and Hunter and Murison [60], respectively. This suggests growth in the popularity of feeding raw meat diets. This is despite low support among veterinarians, health warnings against them, and lack of evidence supporting their use [62]. It may also be the case that guardians feeding raw meat were overrepresented in this survey. It must be noted that purposive sampling was undertaken for vegan guardians, so they *are* likely overrepresented, which would over-accentuate the downward tendency in conventional meat-based diets. For instance, without purposive sampling, the survey of primarily American and British pet guardians by Dodd et al. [16] found that just 1.6% (48/2940) of dog guardians feed their dogs a vegan diet. Few other estimations of feeding vegan diets exclusively exist to date.

Additionally, home-prepared dog food (raw and cooked) was exclusively provided by 10–14% of guardians in the present study. This is higher than the almost 2% reported by Hunter and Murison [60], but similar to the 7% reported by Dodd et al. [46]. Additionally, only 43% of this 10–14% used a recipe. This study has highlighted the continued growth in raw meat-based dog food and homemade dog food, and so affirms that significant welfare risks regarding these remain due to risks of pathogen contamination [62] and nutritional deficiencies [63]. Similarly, weighing of dog food was practiced by 48%; this would be a feasible way for guardians to help manage dog obesity risks, as would having weighing scales to monitor dog weights.

The study has also mirrored some associations found in the extant literature between certain human and animal demographics and dog diet choice. For instance, this study found income to be negatively correlated with feeding dogs a vegan diet, which mirrors a similar finding by Hunter and Murison [60]. These findings suggest that having a higher income does not automatically lead to a higher likelihood of purchasing vegan dog food. The present study also mirrored the finding by Dodd et al. [16] regarding the feeding of vegan diets to dogs being almost exclusively practiced by vegan guardians. This was expected. For instance, Oven et al. [64] have shown how the 4 N justifications typically used by humans for continuing to consume meat—that meat is natural normal, necessary, and nice—can also apply to justifications for continuing to feed companion animals meat. As vegan guardians are already abstaining from all animal product consumption themselves, they may be less likely to have these justifications already prepared in relation to their companion animals’ consumption of meat.

Novel associations were found as well with regard to human demographics, particularly in relation to guardian gender and working in the vet/pet industries. First, male guardians were 148% more likely to feed their dogs a vegan diet compared to females. The same result was found regarding cat guardians in a forthcoming study by Mace et al. [51]. Additionally, treatments with less conventional support have also likewise been found to have greater uptake by male guardians of other species too (e.g., chickens [65]). This could be due to higher tendencies toward assertive behavior in males [66], despite numerous studies indicating greater support for animal welfare, veganism, and environmental sustainability among females relative to males [67]. Second, guardians working in the vet/pet industries were 49% less likely to feed a vegan diet. This is unsurprising as the British Veterinary Association has only recently issued positive (albeit cautious) advice regarding feeding dogs vegan diets [43], with most other veterinary bodies across the world not yet having adopted such positions.

In terms of dog demographics, dogs progressing onto a medical diet were 61% less likely to be fed a vegan diet prior to the medical diet. This indicates the healthfulness of vegan diets as guardians feeding vegan diets were less likely to need to switch to medical diets. However, it could also reflect a lack of vegan prescription diets—making guardians committed to vegan diets less likely to switch when dogs become ill and are prescribed such diets.

In the present study, the two oldest age groups (60–69 and 70+) were less likely to feed their dogs vegan diets relative to the 18–29 age group. The authors are unaware of other extant data based on tests of associations between these variables. However, older age groups are often less open to new ideas and learning [68], so this could explain the finding. Other Europeans and North Americans were also significantly *more* likely to feed their dogs a vegan diet than guardians in the UK. Vegan pet food markets are known to be slightly bigger in the US and Europe [69], which could explain these results. Additionally, *intra*national differences in pet diet choice have been found, such as in the UK [60], which could be investigated further in future research. Due to the UK vegan pet food market’s forecasted second fastest compound annual growth rate from 2025 to 2035 [70], such regional differences could be expected to change or neutralize.

Regarding purchasing determinants, this study found the top five determinants for current diets to be health/nutrition, diet quality, palatability, naturalness, and price. Few extant relevant studies agree on the top determinants for contemporary dog food purchasing decisions. For instance, brand reputation [71], nutritional benefits [45,72], palatability [73], pet preference [47] and price [74] have each been demonstrated as the most important determinant in different surveys. Of note is that the studies by Arachchige et al. [71] and Schleicher et al. [45] did not differentiate between dog and cat owners. The other top determinants (after those ranking first) also differed in the order they were ranked by respondents. For instance, in the studies by Simonsen et al. [74], Schleicher et al. [45], and Hunter and Murison [60], ‘ingredients used’ featured among the top factors while ‘ingredients’ were not a key motivator in Naughton et al.’s [47] study (it was not listed as an option in the current study). Cost is another example; it did not feature as a top factor in the study by Naughton et al. [47], but was rated as of ‘slight importance’ in Schleicher et al.’s study [45], which potentially is aligned with its fifth ranking position in the present study. Cost *was* rated as the top consideration by Simonsen et al. [74], but only five choices were given in that study, and 11 years had passed since its publication, at the time of writing. Hunter and Murison [60] also found that recommendations were a leading purchasing determinant, but this ranked 10th in the present study. In the largest number of responses generated by a survey of this kind to date (n = 33,562), though not peer-reviewed, McCormick [72] uniquely found prior experience to be a key determinant (alongside dog health as per other studies). Naturalness has also been found to be important in other studies (e.g., Dodd et al. [16]; Silva et al. [75]). The absence of environmental credentials featuring among the most important determinants in the present study echoes findings of a small survey-based study in the Netherlands [76]. Future research should streamline the terms used surrounding purchasing determinants to enable better comparisons.

Categorized purchasing determinants were tested for associations with human and dog demographics. The impacts of guardian diet, income, education levels, and dog diet were most noteworthy. First, tendencies were found regarding *decreasing* animal consumption and: (a) *decreasing* importance of Pet Focus I (i.e., health and nutrition, palatability, and diet quality), Pet Focus II (i.e., diet naturalness, freshness, and reputation), and Personal Focus [only price/convenience subitems]—to the point of significance for vegans in each category (relative to omnivores); and (b) *increasing* importance of Personal Values—to the point of significance for all dietary types in all categories (relative to omnivores). Care should be taken here to avoid the incorrect assumption that guardians reducing or eliminating animals from their diets find dog-focused aspects—particularly those relating to Pet Focus I as they are more intrinsically related to dog welfare—less important than omnivores when purchasing dog food. Crucially, the results demonstrate this, as Supplementary Figure S2 shows how the health/nutrition subitem of Pet Focus I is actually rated slightly higher by reducetarians, vegetarians, and vegans, than by omnivore guardians. The results could instead relate to more knowledge concerning the pet food industry. This could include knowing that there are minimum quality standards pet foods need to reach (e.g., in the UK) in order to be marketed as nutritionally complete, and an awareness of alternative dog foods that exist that better align with personal values. Thus, it could mean that such extra knowledge could result in a greater sense of freedom to prioritize pet food that aligns with personal values. In the case of vegans, who will likely have a heightened awareness of the prescribed ‘killability’ [77] of farmed animals compared to dogs, and the drastically different living conditions of well-cared for dogs compared to most farmed animals, it could also be simply that their values of fair treatment to all sentient beings is prioritized first, but this does not mean dog health, nutrition, and preference, is unimportant.

Aside from guardian diet, a tendency was found regarding increased valuing of Pet Focus I and Personal Values with increasing education levels achieved. Similar results were found by Rombach and Dean [78]. Unsurprisingly, reduced importance of Personal Focus was found with increasing guardian age and increasing income. Generally, there may be more spare funds and time as guardians age increases [79], which can explain these tendencies that are also corroborated by Rombach & Dean [78].

Finally, guardians already feeding their dogs a vegan diet rated all purchasing determinant categories (except Personal Values) significantly less important than those feeding a conventional meat-based diet, with the reverse being the case for Personal Values. Given that it is almost exclusively vegan guardians who *currently* feed their dogs vegan diets, this is unsurprising as it matches the aforementioned associations found in relation to guardian diet. Interestingly, while guardians feeding raw meat-based food similarly rated Personal Focus less important and Personal Values more important than those feeding a conventional meat-based diet, in contrast to those feeding a vegan diet, they rated Pet Focus I and Pet Focus II *more* important than those feeding a conventional meat-based diet. With regard to Pet Focus II, this was particularly in relation to the subitems naturalness and freshness (not reputation). This aligns with prior studies that have found guardians feeding raw meat to particularly value naturalness and freshness (e.g., Craig [80]; Lyu et al. [62]; Serra-Castelló et al. [81]).

### 4.2. Acceptance and Essential Characteristics of More Sustainable Dog Diets

Nearly half (43%) of all dog guardians found at least one of the more sustainable alternatives listed acceptable, while 56.8% (1231/2169) found none of the provided alternatives acceptable. Cultivated meat-based pet food was the most accepted alternative (selected by 24%), while vegetarian, insect-based, and vegan pet food options were selected by 17%, 16%, and 13% of respondents, respectively. The true figure may actually be higher for selection of vegan pet food. This is because there were also fungi-based and algal-based options listed (each selected by 7% of respondents), and it is unknown if there were any respondents selecting fungi- and algae-based options who did not select vegan pet food.

Guardian diet was the human demographic factor most strongly associated with acceptance of alternatives. Acceptance of vegan dog food among vegan guardians was 55%, thus clearly at a much higher rate than among nonvegan guardians. Indeed, strong tendencies were found regarding the acceptance of all alternatives (except insect-based diets) increasing in line with decreasing animal consumption. We did not enquire about any reservations regarding certain alternatives. However, the aforementioned clear tendency (other than for insect-based dog food) could be due to the continued intensive farming of potentially sentient animals that insect-based dog food necessitates, and its questionable environmental footprint [24,82,83]. Indeed, belief in insect sentience has been found to reduce the acceptability of insect-based dog food in a recent UK-based survey [84]. Nevertheless, insect-based dog food was still generally considered slightly more acceptable by guardians within all other dietary categories, relative to omnivores. Also, with regard to the tendency of increasing acceptance of vegetarian dog diets with reducing animal consumption, of note was the slight dip in this tendency in terms of vegan guardians’ rating of vegetarian dog food. This is unsurprising due to the welfare and environmental problems vegans recognize as associated with vegetarian diets.

Other studies have found higher rates of acceptance of vegan pet food. For instance, in an international survey of 3673 pet guardians by Dodd et al. [16], 35% of all pet owners were open to transitioning to plant-based (vegan) pet food (versus 13% in this study), including 78% of vegan guardians (versus 55% in this study), though more assurances regarding the nutritional adequacy of the food was required before they would switch. However, Dodd et al. [16] did not distinguish between dog and cat guardians. In a survey of 1000 general dog/cat guardians in the UK, The Vegan Society [85] found a similar figure: 32% of 500 dog guardians were open to transitioning their dogs to a vegan diet and this hinged on the healthfulness of the diet. The reason for the differences in acceptance rates between the present study and other extant studies is unclear. However, the markets are larger in the areas focused on in other studies, which is likely fostering more uptake and support [69,70].

Other studies have confirmed that cultivated meat-based pet food is the most accepted alternative among pet guardians, and have shown even higher rates of acceptance than the present study [64]. However, it should be noted that microbial fermentation-based pet food had not yet emerged at the time of questionnaire design, and this is now another option to be added to the alternatives listed in surveys, with products already available [26].

There were also interesting tendencies regarding a few other demographic variables. Most notably, these included: (a) increased acceptance of all alternatives (except animal-free) with increasing education, and (b) Europe and Oceania being more accepting of animal-free and cultivated meat-based dog food relative to UK guardians. Regarding the former point, it was surprising that 100% animal-free dog food did not also increase in acceptance (like the other alternatives) with increasing guardian educational levels—this demonstrates that educational work still needs to be done focusing on nutritionally sound vegan diets for dogs.

Dog demographics also had an impact. Most notably, guardians currently feeding raw meat-based food had reduced acceptance of all alternatives relative to those feeding conventional meat-based diets. Additionally, both increasing dog age and guardians of dogs progressing on to medical diets led to greater acceptance of animal-free dog diets—potentially, certain health improvements arising from feeding dogs vegan diets [40] are beginning to reach some dog guardians, which may explain these results. Finally, guardians of sexually intact dogs were less accepting of all alternatives. Again, this is an expected result as guardians of sexually intact dogs are particularly associated with the raw meat movements and tendencies toward notions of naturalness [44,86].

The top five essential characteristics that alternatives would need to offer to be chosen by guardians currently feeding conventional or raw meat-based diets were: nutritional soundness, pet health assurances, good quality, palatability, and naturalness. Relative to current purchasing determinants discussed in the previous subsection, these essential characteristics were broadly similar. However, price was demoted to sixth position, while environmental credentials moved up slightly to seventh position from eighth. However, it should be noted that health and nutritional characteristics were combined into one option for current purchasing determinants, while they were separated into two for essential characteristics of alternative dog foods. This means that the extent of the demotion of price and promotion of environmental sustainability may have been slightly masked. If still combined, price could remain in fifth position, and environmental sustainability could reach sixth position (albeit still out of the top five). The authors are unaware of other extant studies that have surveyed dog guardians regarding characteristics they find essential when considering purchasing and feeding their dog an alternative more sustainable diet.

When categorizing the essential characteristics of alternatives, *decreasing* consumption of animals and *increasing* education levels both had positive impacts on the importance of all categories. For current purchasing determinants, this was true of fewer categories (only Personal Values for decreasing animal consumption and only Pet Focus I and Personal Values for educational levels). Because the question about essential attributes of alternative dog foods was only for those currently feeding conventional or raw meat-based dog food, it could be that the absence of those already feeding a more sustainable dog diet further accentuated the importance of these additional categories—both because the alternatives would be more novel to the respondents and because the respondents may be less motivated regarding the need for them. All categories also reduced in importance with increasing guardian age (this was not found for current purchasing determinants—just Personal Focus). It is unclear why this would be—potentially becoming habituated in one’s ways. There was also increased importance of all categories among Other Europeans and those from Oceania, relative to those from the UK. Again, it is unclear as to why, especially given the larger market size of alternatives elsewhere in Europe as discussed earlier.

Guardians feeding raw meat-based dog food and of sexually intact dogs rated all categories less important relative to guardians feeding conventional meat-based dog food and of neutered dogs, respectively. This included particularly low ratings of the subitem reputation, and excluded the subitem of freshness. This is aligned with discussions in previous and subsequent subsections regarding mistrust of norms and of valuing naturalness among these guardians [62,86].

### 4.3. Dog Diet Information Sources

The top five information sources used by dog guardians for dog diet decisions were, in order, labels/packaging, scientific articles/textbooks, business webpages, veterinarians, and special interest groups. It is difficult to directly compare these findings with others in the extant literature as different numbers and types of choices are often offered to respondents. Schleicher et al. [45] found that while veterinarians were the most influential in guiding guardians’ pet food choices, social media, non-veterinary websites, and peers were growing in their influence. Similarly, Hunter and Murison [60] found veterinarians to be ranked second as a source of nutritional information, while online (websites) were ranked first; however, the list provided to respondents did not include labels/packaging, for instance, as the present study did.

When the information sources were categorized, all categories increased in importance with decreasing animal consumption (especially for the Media/Literature category and except for Vet/Pet Care) and with increasing education levels (except Product-Specific). This could be explained by heightened knowledge and awareness of both intensive farming harms, and controversies within the pet food industry, that often accompany reduced animal product consumption. Increased educational levels among guardians may increase awareness about the importance of trustworthy (and potentially varied) sources of information.

Tendencies of reductions in the importance of all categories were found with increasing guardian age—except for Vet/Pet Care, though two higher age groups (50–59 and 60–69) still demonstrated significantly lower valuation of this category relative to the 18–29 age group. This overall tendency could be due to increasing confidence and/or habit-formation in dog care as guardians aged. Some guardian gender differences were also found insofar as male guardians were less likely to use social media than female guardians. Gender differences in social media use—for instance, lower male use of Pinterest, Snapchat, Instagram, TikTok, and Facebook—have been reported in a recent Ofcom online report [87]. Unsurprisingly, those working in the vet/pet industries were more likely to use Media/Literature category (especially scientific literature) and less likely to use Social Media relative to those not working in such industries. Guardians from all regions also used the category Media/Literature more than those from the UK. The reasons for this were unclear.

Finally, a significantly reduced rate of importance of Vet/Pet Care by vegan guardians was found, particularly in relation to paraprofessionals. This is further reflected in the fact that guardians already feeding vegan rate Vet/Pet Care significantly lower than those feeding a conventional meat-based diet—but again, this mainly applies to nonveterinary professionals, with veterinarians being the only subitem rated highly. This demonstrates that vegan guardians and those feeding a vegan diet still value veterinarian input. This stands in contrast to those feeding a raw meat-based diet who also were found to value the Vet/Pet Care category significantly less than those feeding a conventional meat-based diet, but in their case, the veterinarian subitem was rated particularly low. This mirrors extant findings regarding lower trust in veterinarians by raw meat advocates and guardians feeding raw meat [88].

### 4.4. Limitations

Given the disproportionate representation of female participants, of those with some level of college/university level education, and of residents of the UK in this study, the generalizability of the reported population-level relative frequencies of specific subgroups to broader national populations should be approached with caution. Similarly, the limited number of dog guardians from non-European regions necessitates careful interpretation of results pertaining to those areas. For instance, different cultures may differ in their level of acceptance of cultivated meat and nutritionally sound recipes used for pet food. The sample also included a substantially higher proportion of vegans compared to the general population. This was achieved deliberately to obtain robust and meaningful data from this subgroup; however, it may limit the applicability of the reported subgroup frequencies to the wider population. All associations reported in this work were estimated using regression models. As regression is a conditional analysis, the overrepresentation of the aforementioned subgroups was not expected to substantially bias these findings. The results may not be fully generalizable to specific subgroups, such as guardians of dogs with medical conditions or those involved in breeding, underscoring the need for targeted research within these specialist populations. It is also important to note that respondents with dogs maintained on medical diets were instructed to answer based on their dogs’ experiences in the year prior to starting the medical diet. This should be considered when interpreting data from this cohort. Furthermore, the questionnaire referred only to a ‘vegan diet’ rather than specifying ‘nutritionally sound vegan diets.’ The use of this more precise terminology, which is increasingly prevalent in the literature (e.g., Nicholles & Knight [13]), could potentially increase its acceptance among dog guardians, suggesting that actual acceptance rates may be higher than those observed in this study. The use of a convenience sample relying on self-reported data from English-speaking participants introduces potential biases [89]. These risks were mitigated by recruiting a large sample and employing purposive sampling methods to increase the inclusion of a diverse range of perspectives. Lastly, the goodness-of-fit of our regression models (i.e., the AUC and R^2^ values) was (very) low in some cases. However, as outlined in our Statistical Analysis section, we do not believe that this hindered the interpretation of the estimated effect patterns and significances given our social science research framework.

### 4.5. Recommendations

Based on this study and others, health attributes of dog food are the most important purchasing determinants affecting both current diets and potential purchasing of more sustainable alternatives. Thus, it is important for manufacturers of such alternative diets to continue efforts to gain endorsements from national and international dog/pet institutions, confirming the suitability of nutritionally sound vegan, cultivated meat, and microbial protein-based diets for safeguarding dog health. Complementary to this is the need for continuing with outreach work to raise awareness among the public. Additionally, while price does not feature among the most important purchasing determinants of more sustainable dog food, it is *not* insignificant; thus, producers should still factor it into considerations.

Dog guardians who have committed to some form of reduction or elimination of animal products will likely be the most receptive to alternative more sustainable dog food options. Additionally, male guardians may be particularly receptive to feeding nutritionally sound vegan diets, at least before wider social acceptance; however, this does need further corroboration given the low representation of male guardians among the respondents. In contrast, dog guardians currently feeding raw meat may be a particularly difficult target group for encouraging adoption of more sustainable alternative dog diets. While wise to focus on those guardians most receptive, it could also help in outreach work to focus on natural, fresh, and healthy aspects of alternative diets, where applicable. As many dog guardians who feed conventional meat-based dog food also value naturalness, highlighting the *un*natural aspects of conventional meat-based dog food could help facilitate guardians’ transitions to more sustainable dog diet choices.

Veterinarians can be assured that most clients will still be wishing to safeguard their dogs’ health and nutritional needs when considering alternative more sustainable dog food options. Veterinarians should ensure they are aware of the latest evidence in this field and assist by sharing this knowledge with these clients.

Cultivated meat should also be prioritized due to dog guardians being most receptive toward this alternative. Future research should incorporate microbial protein-based dog diets and reducetarian intentions. It should also seek to synchronize language and terms used on survey options to enhance comparability between studies.

## 5. Conclusions

The global dog food industry was valued at USD 41.4 billion in 2022. It is also forecasted to grow by 5.1% annually up to 2030. Among this projected growth is an array of alternatives to standard commercial meat-based dog food, such as those based on nutritionally sound vegan, cultivated meat, microbial protein, insect and raw meat ingredients. Motivations for feeding alternative dog foods can include superior health outcomes, improved sustainability, and/or reduced ethical concerns surrounding the use of ‘food’ animals. However, concerns in some of these areas remain for diets based on insects or raw meat.

This study aimed to increase understanding of dog food purchasing determinants, dogs feeding patterns, factors affecting the acceptance of more sustainable alternative dog diets, and information sources used by guardians for dog diet decisions. The study centers on the results from an online survey (n = 2596). The principal findings indicated that 84% (2188/2596) of participants currently provided their dogs with either conventional or raw meat-based diets. Of these 2188 respondents, 2169 provided information regarding alternative more sustainable dog diets that they would find acceptable. Over 43% (938/2169) considered at least one of the more sustainable alternative dog food options to be acceptable. These 938 respondents—and 10 others who deemed all options unacceptable, but also simultaneously selected one of the alternatives as acceptable—provided information about characteristics alternative diets would need to provide, in order to be chosen. The majority (85%, 801/948) identified nutritional adequacy of the product as the most influential factor in their purchasing decisions. Among the alternative options provided, cultivated meat-based dog food was the most favored (24%, 529/2169), followed by vegetarian (17%, 359/2169), insect-based (16%, 336/2169), and vegan (13%, 290/2169) diets. Among all survey respondents, the three most frequently utilized sources of information for making decisions regarding dog diets were product labels and packaging (42%, 1080/2596), scientific literature (38%, 989/2596), and business websites (35%, 900/2596).

Across all examined human and canine demographic variables, both the human and dog diets exhibited the strongest and most consistent associations with current feeding practices, acceptance of alternative diets, and the selection of information sources. Notably, greater reductions in animal product consumption were usually linked to more positive outcomes in these areas. Key recommendations include the need for more endorsements from dog/pet health institutions for encouraging greater confidence in the healthfulness of more sustainable dog diets. Future research should incorporate pet food based on microbial protein as another more sustainable alternative. Cultivated meat-based dog food remains the most popular sustainable alternative.

## Figures and Tables

**Table 1:** Distribution of dog guardian demographic characteristics (n = 2596 in each category).

	Percentage	Frequency
CONTINENTAL REGION		
UK	71.6%	1858
Other European	15.0%	389
North America	5.7%	149
Australia/New Zealand/Oceania	4.5%	116
South America	1.7%	43
Asia	0.9%	24
Other	0.4%	10
Africa	0.3%	7
		
**HIGHEST EDUCATIONAL LEVEL**		
Did not complete high school	1.7%	43
High school or equivalent	18.3%	474
College or University award < undergrad	28.7%	745
University undergraduate degree	28.3%	735
Postgrad < doctorate	19.6%	508
Doctoral degree	3.5%	91
		
**AGE CATEGORY**		
18–19	0.7%	17
20–29	15.0%	389
30–39	21.0%	546
40–49	20.6%	536
50–59	23.0%	596
60–69	15.4%	401
70+	4.3%	111
		
**HUMAN DIET**		
Omnivore	40.2%	1044
Reducetarian	21.6%	562
Pescatarian	5.1%	133
Vegetarian	10.1%	263
Vegan	22.1%	574
Other	0.8%	20

**Table 2:** Distribution of current dog diets (n = 2596) and acceptance of more sustainable alternative diets among those (n = 2169) currently feeding conventional/raw meat-based dog food.

	Percentage	Frequency
CURRENT DOG DIET		
Meat-based—conventional	52.3%	1359
Meat-based—raw	31.9%	829
Vegan	12.8%	333
Vegetarian	1.3%	34
Mixture	0.7%	17
Unsure	0.4%	10
Meat-based—cultivated	0.3%	7
Insect-based	0.2%	6
Fungi-based	0.0%	1
		
**ACCEPTANCE OF MORE SUSTAINABLE DOG DIET**		
None of the options	56.8%	1231
Meat-based—cultivated	24.4%	529
Vegetarian	16.6%	359
Insect-based	15.5%	336
Vegan	13.4%	290
Fungi-based	7.1%	153
Algae-based	6.7%	146

Note: For ‘Acceptance of more sustainable diet,’ 2169 guardians responded from a possible 2188. Multiple responses were possible, thus the percentages and frequencies represent the proportion of all respondents selecting each answer option. Whilst fungi- and algae-based dog foods are vegan, the options listed reflect those offered to respondents within this survey. Respondents could select more than one option when answering this question concerning alternative options. “None of the options” was an answer option provided to participants who did not find any of the listed alternatives acceptable.

**Table 3:** Distribution of source of dog food purchases, type of dog food used, and percentage of commercial dog food used (n = 2596 in each category).

	Percentage	Frequency
SOURCE OF MAJORITY OF DOG FOOD		
Direct from the manufacturer	25.6%	664
Ordered online	25.0%	648
Pet store	23.9%	620
Other store	11.0%	285
Diet is 50% or more homemade	9.8%	254
Other	2.5%	66
Veterinary clinic	2.3%	59
		
**DOG FOOD CONSISTENCY**		
Commercial dry kibble	37.6%	976
Commercial raw	24.1%	626
An equal mix of dry food, with moist or raw	14.7%	382
Commercial canned/pouch	6.9%	178
Home-prepared cooked	5.7%	149
Home-prepared raw	5.2%	136
Commercial dry premix (for use with additional items)	2.4%	62
Other	2.0%	51
Human food	1.4%	36
		
**% COMMERCIAL**		
100%	24.8%	643
75–99%	46.3%	1202
50–74%	12.4%	322
25–49%	4.9%	126
5–24%	3.9%	101
0–4%	7.8%	202

**Table 4:** Distribution of respondents’ use of dog treats (n = 2371 in each category).

	Percentage	Frequency
FREQUENCY OF SNACKS/TREATS		
More than once a day	48.9%	1160
Once a day	34.5%	817
More than once a week but less than once a day	13.8%	327
Once a week	1.7%	41
Less than once a week	1.1%	26
		
**TYPE OF TREAT**		
Vegetables or fruit	56.6%	1342
Other commercial treats	50.5%	1197
Dental/oral bars or chewable sticks	48.7%	1154
Human food prepared at home	38.8%	921
Raw meat or bones	32.1%	762
Other	10.4%	246
Human food from other sources	8.3%	197

Note: Multiple responses were possible when selecting ‘type of treat.’ Thus, the percentages and counts represent the proportion of all respondents selecting each answer option. ‘Other’ (10.4%, n = 246) commonly comprised details of specific commercial treats given (e.g., rabbit ears or vegan chews) or specific homemade treats given (e.g., liver cake, dehydrated meat-based snacks, or vegan dog biscuits).

**Table 5:** Distribution of veterinary nutritional advice received (n = 384).

	Frequency
ADVICE	
Senior food/prescription/food for specific ailment	53
Particular food brands (e.g., Royal Canin, Hill’s, Chappie—often what veterinarians stock).	47
Add supplements (e.g., fiber, B vitamins, ‘good’ fats, calcium)	46
Reduce intake, implement weight controls, reduce fat, portion controls	44
Hypoallergenic diet	37
Kibble (often what veterinarians stock)	29
Against raw	28
Elimination diet	17
Raw	17
Bland/simple diet (often based around fish, chicken, rice)	17
Miscellaneous (blood tests, reduce crude ash ratio, tailor-made recipes, no sugar, low salt, low milk, no apple/banana, pH test of urine, follow Chinese Medicine, do not alter food as it is a genetic problem)	12
Commercial, balanced dog food (often what veterinarians stock)	10
Vegan is acceptable	10
Own research as I am a vet	8
Grain-free	7
Against vegan	7
Moderate/low protein	6
Tips to increase eating and weight	6
Avoid grain-free	5
Home-cooked vegan food	4
Increase protein	4
Specific ratios of protein, fats, carbs	3
Hydrolyzed food	3
Against particular brands (Acana Orijen)	2
Add egg/yogurt	2
Premium dog foods	2
Against home-cooked	1
Avoid standard commercial foods	1

Note: Respondents were able to state multiple recommendations. Text in parentheses comprises either examples or extra information to help understand the advice category.

## Data Availability

This study’s data, questionnaire, and the R code used for the data’s statistical analysis are available at https://osf.io/nbepu (accessed 14 October 2025).

## Supplementary Materials

The following supporting information can be downloaded at https://www.mdpi.com/article/10.3390/ani15202988/s1. Figures S1–S7; Tables S1–S3.
