# Supplementary material for: Consumer Acceptance of Sustainable Dog Diets: A Survey of 2639 Dog Guardians
Source: Animals (Basel). 2025 Oct 15;15(20):2988. doi: 10.3390/ani15202988 (PMC12560912; doi:10.3390/ani15202988)
Supplement: Supplementary file 1 [file animals-15-02988-s001.zip › animals-3845815-Supplementary Figures.pdf]

Supplementary Figures

Part 1 – Current diets

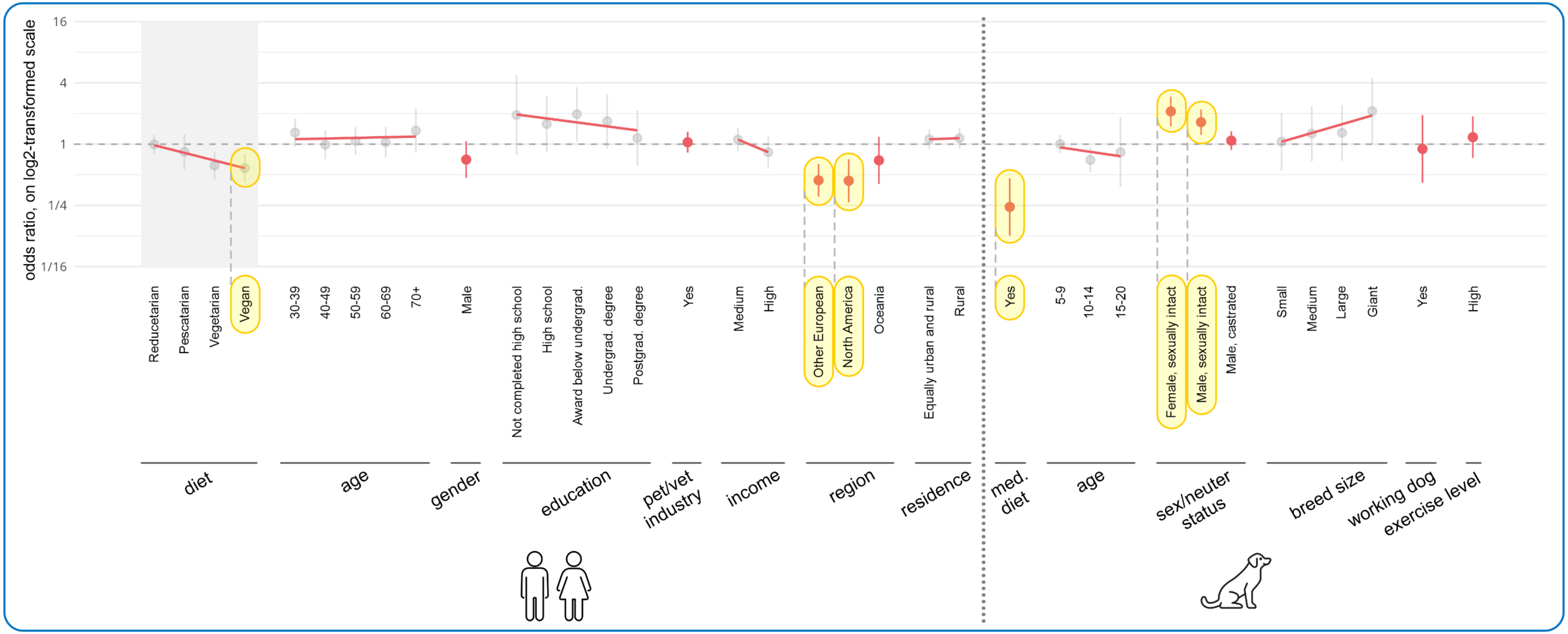

Figure S1. Logistic regression results on the associations between human/dog demographic characteristics and the likelihood of currently feeding raw meat-based diets to dogs (as opposed to conventional meat-based diets), relative to the reference characteristics for each group.

Note: The reference characteristics for these humans and dogs were respectively: (human) aged 18–29, female, doctoral degree, not in pet/vet industry, low income, UK- and urban-based; (dog) no medical diet, aged 0–4, female, spayed, mostly indoors. Effects are depicted as odds ratios, including 95% confidence intervals (not corrected for multiple testing). Effects that are significant after multiple testing correction are highlighted with yellow bubbles. Individual estimated effects of ordinal variables are grayed out in favor of an additional linear trend line. Effects of human diet are highlighted through a gray-shaded area to reflect their separate estimation scheme (i.e., these effects were estimated while not controlling for further human demographic variables) as outlined in section 2.2.

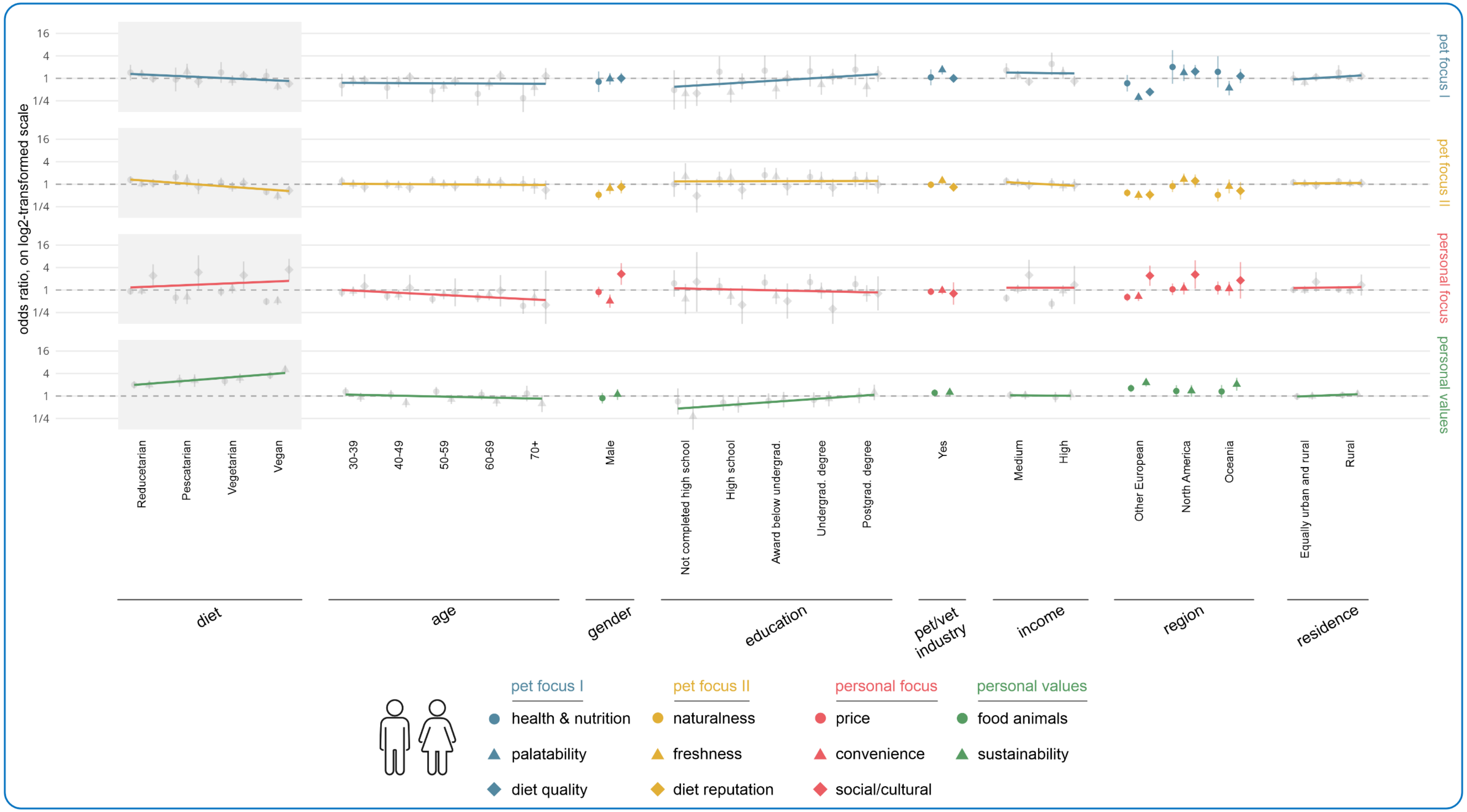

Figure S2. Logistic regression results on the associations between human demographic characteristics and current dog food purchasing determinants, relative to the reference characteristics for each group.

Note: This figure relates to Figure 7 from the main paper. It illustrates the results regarding the individual items that comprise the right y-axis categories. The reference characteristics for these humans were: omnivore, aged 18–29, female, doctoral degree, not in pet/vet industry, low income, UK-based, and urban-based. Effects are depicted as odds ratios, including 95% confidence intervals. Due to the exploratory character of this analysis, no multiple testing correction was applied and statistical significance was not investigated. Individual estimated effects of ordinal variables are grayed out in favor of an additional linear trend line. Effects of human diet are highlighted through gray-shaded areas to reflect their separate estimation scheme (i.e., these effects were estimated while not controlling for further human demographic variables) as outlined in section 2.2.

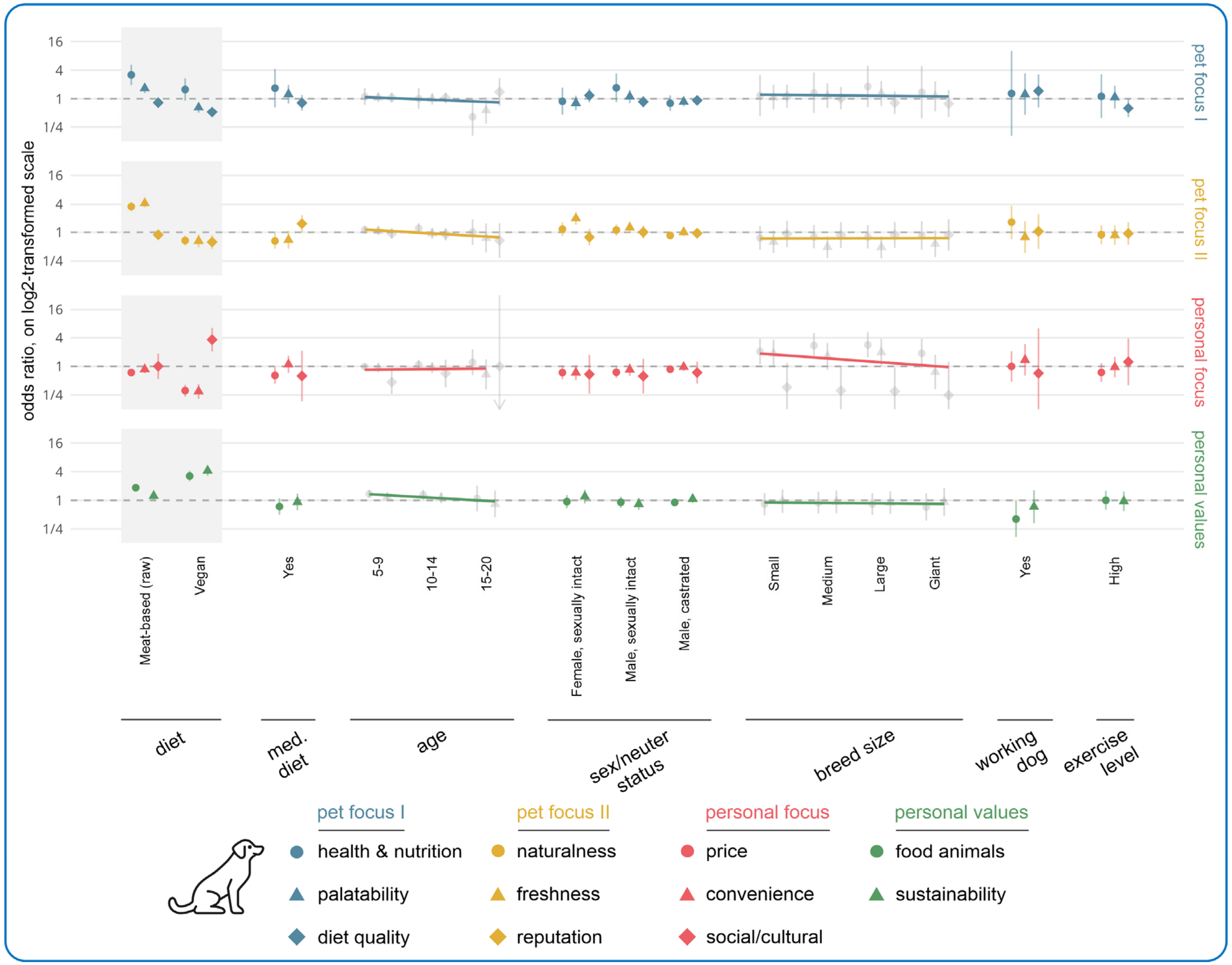

Figure S3. Logistic regression results on the associations between dog demographic characteristics and current dog food purchasing determinants, relative to the reference characteristics for each group.

Note: This figure relates to Figure 7 from the main paper. It illustrates the results regarding the individual items that comprise the right y-axis categories. The reference characteristics for these dogs were: meat-based (conventional); no medical diet; aged 0–4; female, spayed; and mostly indoors. Effects are depicted as odds ratios, including 95% confidence intervals. Due to the exploratory character of this analysis, no multiple testing correction was applied and statistical significance was not investigated. Individual estimated effects of ordinal variables are grayed out in favor of an additional linear trend line. Effects of dog diet are highlighted through gray-shaded areas to reflect their separate estimation scheme (i.e., these effects were estimated while not controlling for further human demographic variables) as outlined in section 2.2.

Part 2 – Alternative diets

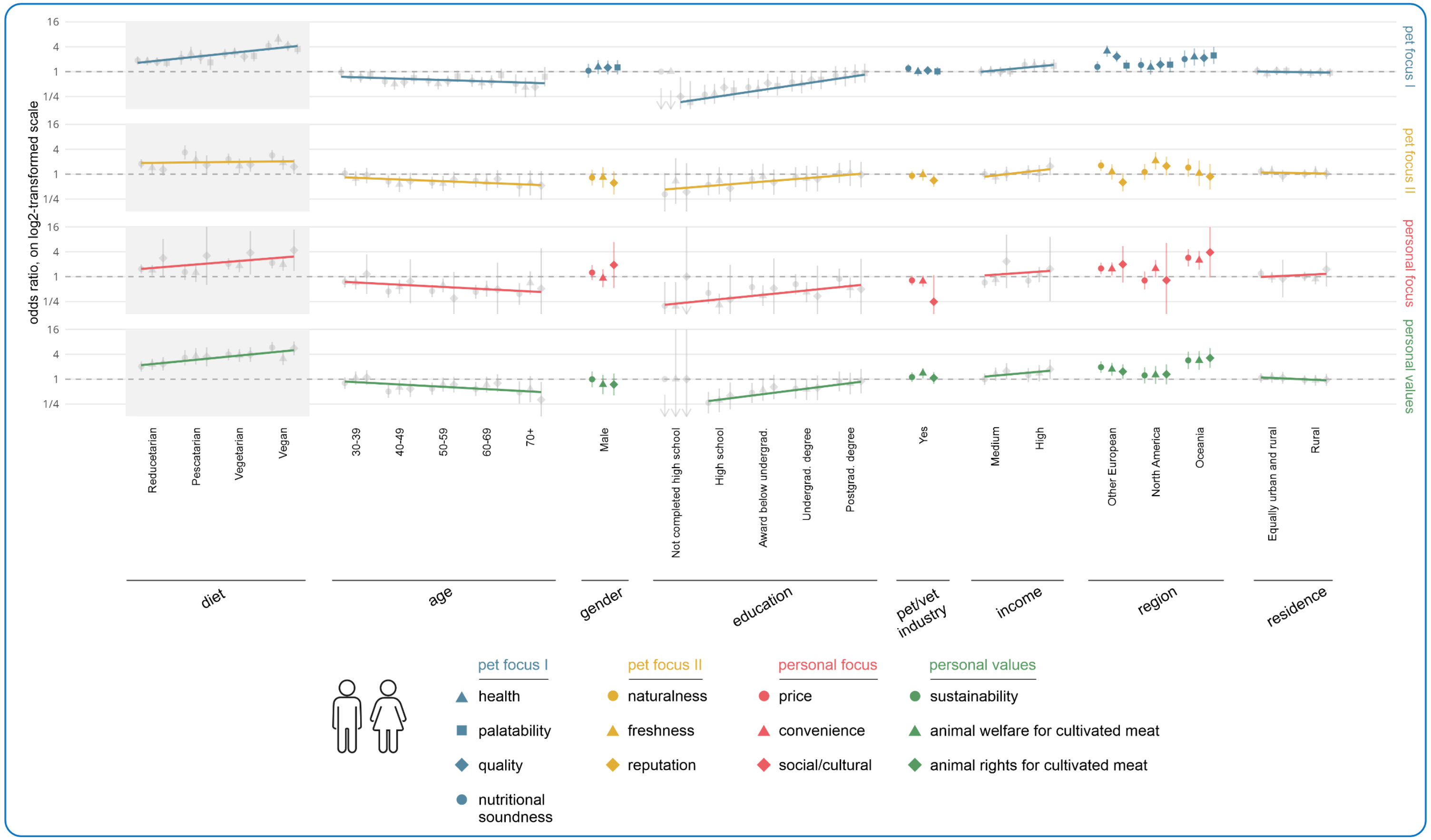

Figure S4. Logistic regression results on the associations between human demographic characteristics and characteristics of more sustainable dog diets considered essential, among guardians currently feeding meat-based dog food (raw or conventional), relative to the reference characteristics for each group.

Note: This figure relates to Figure 11 from the main paper and illustrates the results regarding the individual items that comprise the right y-axis categories. The reference characteristics for these humans were: omnivore, aged 18–29, female, doctoral degree, not in pet/vet industry, low income, UK-based, and urban-based. Effects are depicted as odds ratios, including 95% confidence intervals. Due to the exploratory character of this analysis, no multiple testing correction was applied and statistical significance was not investigated. Individual estimated effects of ordinal variables are grayed out in favor of an additional linear trend line. Effects of human diet are highlighted through gray-shaded areas to reflect their separate estimation scheme (i.e., these effects were estimated while not controlling for further human demographic variables) as outlined in section 2.2.

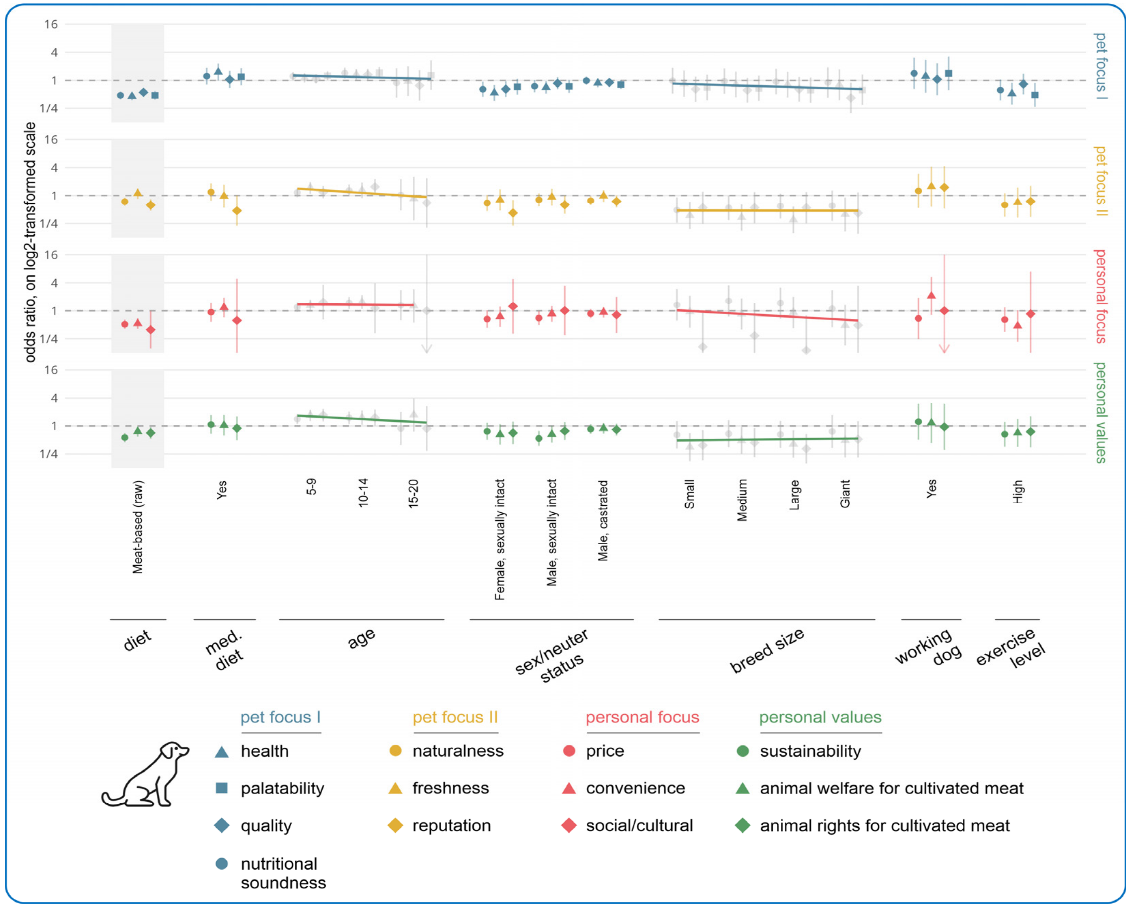

Figure S5. Logistic regression results on the associations between dog demographic characteristics and characteristics of more sustainable dog diets considered essential, among guardians currently feeding meat-based dog food (raw or conventional), relative to the reference characteristics for each group.

Note: This figure relates to Figure 11 from the main paper and illustrates the results regarding the individual items that comprise the right y-axis categories. The reference characteristics for these dogs were: meat-based (conventional); no medical diet; aged 0–4; female, spayed; and mostly indoors. Effects are depicted as odds ratios, including 95% confidence intervals. Due to the exploratory character of this analysis, no multiple testing correction was applied and statistical significance was not investigated. Individual estimated effects of ordinal variables are grayed out in favor of an additional linear trend line. Effects of dog diet are highlighted through gray-shaded areas to reflect their separate estimation scheme (i.e., these effects were estimated while not controlling for further human demographic variables) as outlined in section 2.2.

Part 3 – Information sources

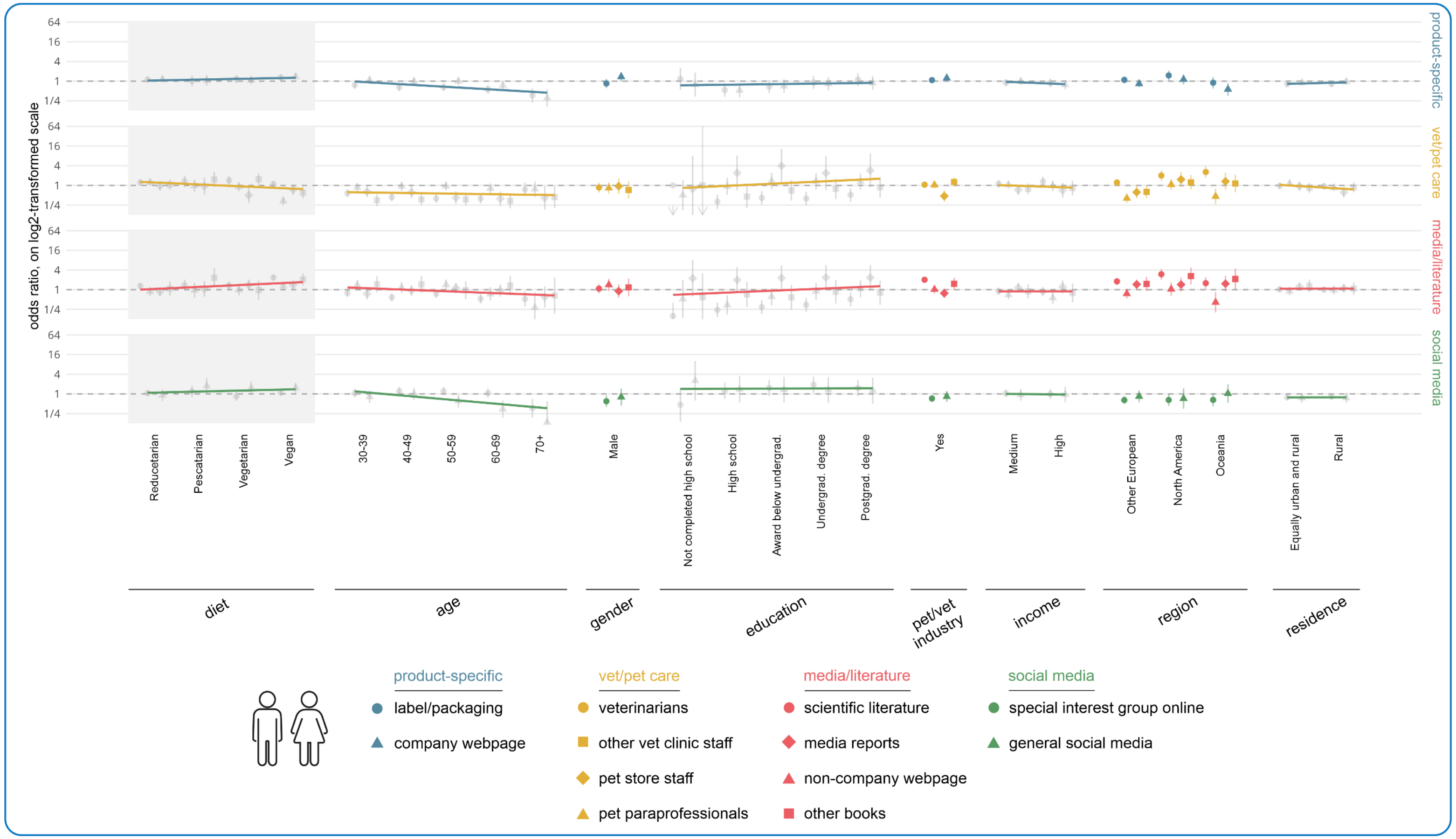

Figure S6. Logistic regression results on the associations between human demographic characteristics and dietary information sources, relative to the reference characteristics for each group.

Note: This figure relates to Figure 13 from the main paper and illustrates the results regarding the individual items that comprise the right y-axis categories. The reference characteristics for these humans were: omnivore, aged 18–29, female, doctoral degree, not in pet/vet industry, low income, UK-based, and urban-based. Effects are depicted as odds ratios, including 95% confidence intervals. Due to the exploratory character of this analysis, no multiple testing correction was applied and statistical significance was not investigated. Individual estimated effects of ordinal variables are grayed out in favor of an additional linear trend line. Effects of human diet are highlighted through gray-shaded areas to reflect their separate estimation scheme (i.e., these effects were estimated while not controlling for further human demographic variables) as outlined in section 2.2.

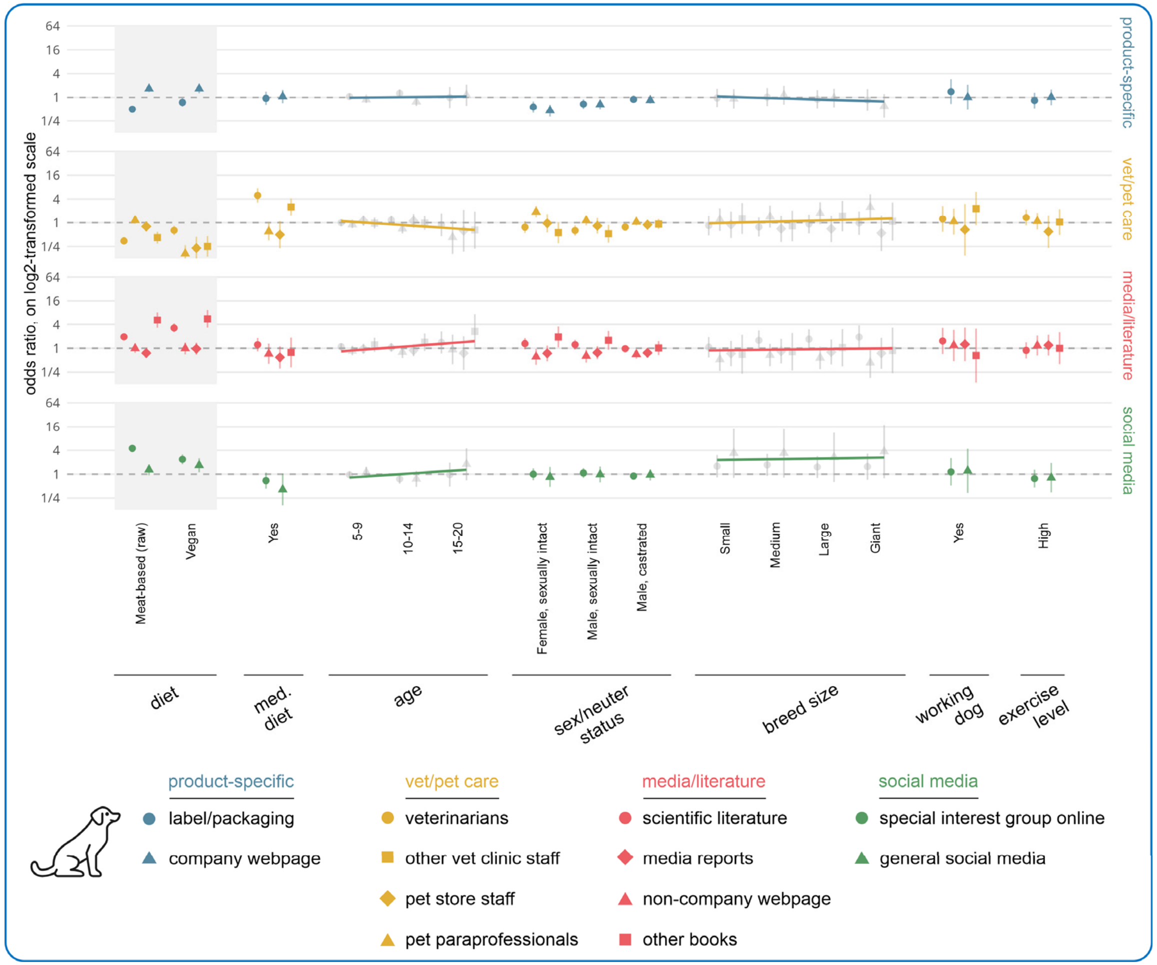

Figure S7. Logistic regression results on the associations between dog demographic characteristics and dietary information sources, relative to the reference characteristics for each group.

Note: This figure relates to Figure 13 from the main paper and illustrates the results regarding the individual items that comprise the right y-axis categories. The reference characteristics for these dogs were: meat-based (conventional); no medical diet; aged 0–4; female, spayed; and mostly indoors. Effects are depicted as odds ratios, including 95% confidence intervals. Due to the exploratory character of this analysis, no multiple testing correction was applied and statistical significance was not investigated. Individual estimated effects of ordinal variables are grayed out in favor of an additional linear trend line. Effects of dog diet are highlighted through gray-shaded areas to reflect their separate estimation scheme (i.e., these effects were estimated while not controlling for further human demographic variables) as outlined in section 2.2.
