# Supplementary material for: Consumer Acceptance of Sustainable Dog Diets: A Survey of 2639 Dog Guardians
Source: Animals (Basel). 2025 Oct 15;15(20):2988. doi: 10.3390/ani15202988 (PMC12560912; doi:10.3390/ani15202988)
Supplement: Supplementary file 1 [file animals-15-02988-s001.zip › animals-3845815-Supplementary Tables.pdf]

Supplementary Tables

Complementing the results section of the main paper, the following five tables summarize the key statistics from Figures 5, 7–9, 11, 13, and S1 regarding correlations between human and dog demographic variables, and key variables of the Research Questions (RQs). There is a table for each RQ. In each table, human demographics are covered first, followed by dog demographics. Additionally, the most noteworthy variables are generally listed first, and with the order of the variable items in the figures also taken into consideration. Significant results (p < 0.05) are boldfaced, with the applicable variables highlighted.

Table S1a. Key statistics relating to RQ1a (current feeding patterns) and Figures 5 and S1.

Note: The data specifically concern Figure 5 regarding likelihood of vegan guardians to feed vegan dog food and Figure S1 regarding likelihood of guardians feeding meat, to feed raw meat-based dog food. Effects are reported as odds ratios, including 95% confidence intervals (not corrected for multiple testing) and p-values corrected for multiple testing.

RQ1a: What feeding patterns exist among dog guardians?		
**Variable item**	**Status cf. reference category**	**Statistics**
**Likelihood among vegan guardians of feeding vegan dog food**		
**HUMAN DEMOGRAPHICS**		
Age: 60-69 years	No trend cf. ages 18-29	OR = -24% (CI: [-71%, +103%], p>0.9999)
Gender: male	Trend cf. females	OR = +148% (CI: [+30%, +374%], p=0.2313)
Pet/vet industry:	Trend cf. not in the pet/vet industry	OR = -49% (CI: [-72%, -4%], p>0.9999)
**Region: Other European**	**Effect cf. UK residents**	**OR = +324% (CI: [+161%, +591%], p<0.0001)**
**Region: North America**	**Effect cf. UK residents**	**OR = +441% (CI: [+129%, +1180%], p=0.0051)**
Education: high school	Trend cf. doctorate	OR = -69% (CI: [-90%, -4%], p>0.9999)
Education: award below undergrad.	Trend cf. doctorate	OR = -72% (CI: [-91%, -19%], p=0.6641)
Income: high income	Trend cf. low income	OR = -67% (CI: [-84%, -31%], p=0.1249)
No/minimal effect: Residence		

DOG DEMOGRAPHICS		
Medical diet	Trend cf. no medical diet	OR = -61% (CI: [-83%, -11%], p=0.8503)
Age: 5-9 years	No trend cf. 0-4 years	OR = +51% (CI: [-6%, +145%], p>0.9999)
Age: 15-20 years	No trend cf. 0-4 years	OR = +7% (CI: [-63%, +210%], p>0.9999)
Breed size: giant breed size	No trend cf. toy size	OR = -55% (CI: [-90%, +101%], p>0.9999)
Exercise level: high	No trend cf. normal exercise level	OR = -51% (CI: [-88%, +101%], p>0.9999)
Sex/neuter status: male, sexually intact	No trend cf. female, spayed	OR = -36% (CI: [-69%, +32%], p>0.9999)
No/minimal effect: Working dog		
**Likelihood among guardians feeding meat, of feeding raw meat**		
**HUMAN DEMOGRAPHICS**		
**Diet: vegan**	**Effect cf. omnivores**	**OR = -42% (CI: [-58%, +20%], p=0.0400)**
Diet: vegetarian	Trend cf. omnivores	OR = -38% (CI: [-55%, -16%], p=0.1006)
Gender: males	No trend cf. females	OR = -30% (CI: [-54%, +7%], p>0.9999)
**Region: Other European**	**Effect cf. UK residents**	**OR = -56% (CI: [-70%, -36%], p=0.0007)**
**Region: North America**	**Effect cf. UK residents**	**OR = -56% (CI: [-73%, -29%], p=0.0376)**
Education: award below undergrad.	Trend cf. doctorate	OR = +97% (CI: [+7%, +264%], p>0.9999)
No/minimal effect: Age, pet/vet industry, income, residence		
**DOG DEMOGRAPHICS**		
**Medical diet**	**Effect cf. no medical diet**	**OR = -76% (CI: [-87%, +54%], p=0.0008)**
**Sex/neuter status: female, sexually intact**	**Effect cf. female, spayed**	**OR = +110% (CI: [+50%, +194%], p=0.0009)**
**Sex/neuter status: male, sexually intact**	**Effect cf. female, spayed**	**OR = +64% (CI: [+23%, +119%], p=0.0323)**
Age: 10-14 years	Trend cf. 0-4 years	OR = -30% (CI: [-47%, -7%], p=0.5495)
Breed size: giant	Trend cf. toy size	OR = +112% (CI: [+1%, +345%], p>0.9999)
No/minimal effect: Working dog, exercise level		

Table S1b. Key statistics relating to RQ1b (current purchasing determinants) and Figure 7.

Note: Starred items in the first column relate to Figure S2. Effects are reported including 95% confidence intervals (not corrected for multiple testing) and p-values corrected for multiple testing. Significant results (after multiple testing correction) are highlighted. P-values are not provided in cases of explorative analyses.

RQ1b: What factors do dog guardians find important when choosing dog diets?			
**Purchasing determinant category / item***	**Variable item**	**Status cf. reference category**	**Statistics**
**HUMAN DEMOGRAPHICS**			
**Pet Focus I**	**Diet: vegan**	**Effect cf. omnivores**	**Estimate = -0.06 (CI: [-0.08, -0.03], p=0.0049)**
**Pet Focus II**	**Diet: vegan**	**Effect cf. omnivores**	**Estimate = -0.10 (CI: [-0.14, -0.07], p<0.0001)**
**Personal Focus**	**Diet: vegan**	**Effect cf. omnivores**	**Estimate = -0.08 (CI: [-0.11, -0.05], p<0.0001)**
Personal Focus	Diet: pescatarian	Trend cf. omnivores	Estimate = -0.06 (CI: [-0.10, -0.01], p>0.9999)
**Personal Values**	**Diet: reducetarian**	**Effect cf. omnivores**	**Estimate = +0.13 (CI: [+0.10, +0.17], p<0.0001)**
**Personal Values**	**Diet: pescatarian**	**Effect cf. omnivores**	**Estimate = +0.19 (CI: [+0.13, +0.26], p<0.0001)**
**Personal Values**	**Diet: vegetarian**	**Effect cf. omnivores**	**Estimate = +0.21 (CI: [+0.16, +0.26], p<0.0001)**
**Personal Values**	**Diet: vegan**	**Effect cf. omnivores**	**Estimate = +0.32 (CI: [+0.28, +0.36], p<0.0001)**
**Pet Focus I**	**Region: Other European**	**Effect cf. UK residents**	**Estimate = -0.15 (CI: [-0.18, -0.12], p<0.0001)**
Pet Focus I	Region: North America	Trend cf. UK residents	Estimate = +0.05 (CI: [+0.01, +0.09], p>0.9999)
**Pet Focus II**	**Region: Other European**	**Effect cf. UK residents**	**Estimate = -0.11 (CI: [-0.15, -0.08], p<0.0001)**
Pet Focus II	Region: Oceania	Trend cf. UK residents	Estimate = -0.08 (CI: [-0.14, -0.02], p>0.9999)
Personal Focus	Region: Other European	Trend cf. UK residents	Estimate = -0.05 (CI: [-0.08, -0.02], p=0.3384)
**Personal Values**	**Region: Other European**	**Effect cf. UK residents**	**Estimate = +0.15 (CI: [+0.10, +0.20], p<0.0001)**
Personal Values	Region: North America	Trend cf. UK residents	Estimate = +0.07 (CI: [+0.001, +0.13], p>0.9999)
Personal Values	Region: Oceania	Trend cf. UK residents	Estimate = +0.11 (CI: [+0.03, +0.19], p=0.7455)
Pet Focus I	Age: 50-59 years	Trend cf. 18-29 years	Estimate = -0.05 (CI: [-0.08, -0.02], p=0.3561)
Personal Focus	Age: 40-49 years	Trend cf. 18-29 years	Estimate = -0.05 (CI: [-0.08, -0.01], p>0.9999)
Personal Focus	Age: 50-59 years	Trend cf. 18-29 years	Estimate = -0.06 (CI: [-0.10, -0.03], p=0.0930)
Personal Focus	Age: 60-69 years	Trend cf. 18-29 years	Estimate = -0.05 (CI: [-0.09, -0.02], p>0.9999)

Personal Focus	Age: 70+ years	Trend cf. 18-29 years	Estimate = -0.11 (CI: [-0.17, -0.05], p=0.0608)
Pet Focus II	Gender: male	Trend cf. females	Estimate = -0.08 (CI: [-0.12, -0.03], p=0.2889)
Personal Focus: price*	Gender: male	Explorative tendency cf. females	OR = -11% (CI: [-36%, +23%])
Personal Focus: convenience*	Gender: male	Explorative tendency cf. females	OR = -49% (CI: [-66%, -24%])
Pet Focus I	Education: high school	Trend cf. doctorate	Estimate = -0.16 (CI: [-0.25, -0.06], p=0.1927)
Personal Values	Education: high school	Trend cf. doctorate	Estimate = -0.10 (CI: [-0.19, -0.01], p>0.9999)
Pet Focus I	Pet/vet industry:	Trend cf. not in pet/vet industry	Estimate = +0.03 (CI: [+0.001, +0.05], p>0.9999)
Personal Values	Pet/vet industry:	Trend cf. not in pet/vet industry	Estimate = +0.05 (CI: [+0.01, +0.09], p>0.9999)
Personal Focus	Income: medium	Trend cf. low income	Estimate = -0.03 (CI: [-0.06, -0.0001], p>0.9999)
Personal Focus	Income: high	Trend cf. low income	Estimate = -0.07 (CI: [-0.11, -0.03], p>0.9999)
Personal Focus: price*	Income: high	Explorative tendency cf. low income	OR = -39% (CI: [-52%, -23%])
No/minimal effect: Residence			
**DOG DEMOGRAPHICS**			
**Pet Focus I**	**Diet: vegan**	**Effect cf. conventional meat**	**Estimate = -0.07 (CI: [-0.10, -0.04], p=0.0008)**
**Pet Focus II**	**Diet: vegan**	**Effect cf. conventional meat**	**Estimate = -0.08 (CI: [-0.11, -0.04], p=0.0047)**
**Personal Focus**	**Diet: vegan**	**Effect cf. conventional meat**	**Estimate = -0.13 (CI: [-0.16, -0.10], p<0.0001)**
Personal Focus	Diet: raw meat	Trend cf. conventional meat	Estimate = -0.03 (CI: [-0.06, -0.01], p=0.6322)
**Personal Values**	**Diet: vegan**	**Effect cf. conventional meat**	**Estimate = +0.31 (CI: [+0.26, +0.35], p<0.0001)**
**Personal Values**	**Diet: raw meat**	**Effect cf. conventional meat**	**Estimate = +0.09 (CI: [+0.06, +0.12], p<0.0001)**
Pet Focus I	Diet: raw meat	Trend cf. conventional meat	Estimate = +0.03 (CI: [+0.01, +0.05], p>0.9999)
**Pet Focus II**	**Diet: raw meat**	**Effect cf. conventional meat**	**Estimate = +0.19 (CI: [+0.16, +0.21], p<0.0001)**
Personal Values	Age: 5-9 years	Trend cf. 0-4 years	Estimate = +0.05 (CI: [+0.01, +0.09], p=0.8976)
No/minimal effect: Medical diet, sex/neuter status, breed size, dog role, exercise level.			

Table S2a. Key statistics relating to RQ2a (acceptance of alternative dog foods) and Figures 8 and 9.

Note: Effects are reported as odds ratios, including 95% confidence intervals (not corrected for multiple testing) and p-values corrected for multiple testing. Significant results (after multiple testing correction) are highlighted.

RQ2a: What proportion of guardians feeding conventional or raw meat-based dog diets would realistically accept more sustainable alternatives?			
**Alternative diet**	**Variable item**	**Status cf. reference category**	**Statistics**
**HUMAN DEMOGRAPHICS**			
**100% non-animal: plant-based**	**Diet: vegan**	**Effect cf. omnivores**	**OR = +8662% (CI: [+5216%, +14341%], p<0.0001)**
**100% non-animal: plant-based**	**Diet: vegetarian**	**Effect cf. omnivores**	**OR = +972% (CI: [+538%, +1702%], p<0.0001)**
**100% non-animal: plant-based**	**Diet: pescatarian**	**Effect cf. omnivores**	**OR = +736% (CI: [+346%, +1468%], p<0.0001)**
100% non-animal: plant-based	Diet: reducetarian	Trend cf. omnivores	OR = +136% (CI: [+36%, +309%], p=0.6480)
**100% non-animal: fungi-based**	**Diet: vegan**	**Effect cf. omnivores**	**OR = +375% (CI: [+188%, +684%], p<0.0001)**
**100% non-animal: fungi-based**	**Diet: vegetarian**	**Effect cf. omnivores**	**OR = +322% (CI: [+152%, +605%], p<0.0001)**
100% non-animal: fungi-based	Diet: reducetarian	Trend cf. omnivores	OR = +127% (CI: [+42%, +263%], p=0.1871)
**100% non-animal: algae-based**	**Diet: vegan**	**Effect cf. omnivores**	**OR = +304% (CI: [+145%, +565%], p<0.0001)**
**100% non-animal: algae-based**	**Diet: vegetarian**	**Effect cf. omnivores**	**OR = +241% (CI: [+103%, +472%], p=0.0011)**
100% non-animal: algae-based	Diet: reducetarian	Trend cf. omnivores	OR = +89% (CI: [+18%, +202%], p>0.9999)
**Vegetarian**	**Diet: vegan**	**Effect cf. omnivores**	**OR = +149% (CI: [+68%, +267%], p=0.0015)**
**Vegetarian**	**Diet: vegetarian**	**Effect cf. omnivores**	**OR = +424% (CI: [+268%, +646%], p<0.0001)**
**Vegetarian**	**Diet: pescatarian**	**Effect cf. omnivores**	**OR = +266% (CI: [+131%, +481%], p<0.0001)**
**Vegetarian**	**Diet: reducetarian**	**Effect cf. omnivores**	**OR = +123% (CI: [+63%, +205%], p=0.0001)**
**Cultivated meat**	**Diet: vegan**	**Effect cf. omnivores**	**OR = +184% (CI: [+107%, +289%], p<0.0001)**
**Cultivated meat**	**Diet: vegetarian**	**Effect cf. omnivores**	**OR = +152% (CI: [+83%, +246%], p<0.0001)**

Cultivated meat	Diet: pescatarian	Trend cf. omnivores	OR = +119% (CI: [+44%, +232%], p=0.0749)
**Cultivated meat**	**Diet: reducetarian**	**Effect cf. omnivores**	**OR = +70% (CI: [+32%, +120%], p=0.0119)**
Insect-based	Diet: vegetarian	Trend cf. omnivores	OR = +97% (CI: [+37%, +184%], p=0.0823)
Insect-based	Diet: reducetarian	Trend cf. omnivores	OR = +50% (CI: [+12%, +101%], p>0.9999)
**Cultivated meat**	**Age: 40-49 years**	**Effect cf. 18-29 years**	**OR = -55% (CI: [-68%, -36%], p=0.0020)**
**Cultivated meat**	**Age: 50-59 years**	**Effect cf. 18-29 years**	**OR = -50% (CI: [-64%, -30%], p=0.0183)**
Cultivated meat	Age: 60-69 years	Trend cf. 18-29 years	OR = -44% (CI: [-62%, -18%], p=0.8666)
Cultivated meat	Age: 70+ years	Trend cf. 18-29 years	OR = -56% (CI: [-77%, -16%], p>0.9999)
Insect-based	Age: 50-59 years	Trend cf. 18-29 years	OR = -43% (CI: [-62%, -13%], p>0.9999)
100% non-animal: plant-based	Age: 70+ years	Trend cf. 18-29 years	OR = -86% (CI: [-97%, -38%], p>0.9999)
Vegetarian	Education: high school	Trend cf. doctorate	OR = -55% (CI: [-77%, -15%], p>0.9999)
Vegetarian	Education: award below undergrad.	Trend cf. doctorate	OR = -53% (CI: [-75%, -13%], p>0.9999)
Cultivated meat	Education: no high school	Trend cf. doctorate	OR = -85% (CI: [-96%, -44%], p>0.9999)
**Cultivated meat**	**Education: high school**	**Effect cf. doctorate**	**OR = -75% (CI: [-86%, -55%], p=0.0016)**
Cultivated meat	Education: below under-grad.	Trend cf. doctorate	OR = -58% (CI: [-76%, -27%], p=0.6152)
Cultivated meat	Education: undergrad. de-gree	Trend cf. doctorate	OR = -47% (CI: [-69%, -8%], p>0.9999)
Insect-based	Education: no high school	Trend cf. doctorate	OR = -84% (CI: [-97%, -23%], p>0.9999)
**Insect-based**	**Education: high school**	**Effect cf. doctorate**	**OR = -83% (CI: [-91%, -65%], p=0.0003)**
Insect-based	Education: below under-grad.	Trend cf. doctorate	OR = -65% (CI: [-81%, -35%], p=0.2677)
Insect-based	Education: undergrad. de-gree	Trend cf. doctorate	OR = -49% (CI: [-72%, -8%], p>0.9999)
**100% non-animal: fungi-based**	**Education: high school**	**Effect cf. doctorate**	**OR = -88% (CI: [-95%, -70%], p=0.0025)**
100% non-animal: fungi-based	Education: below under-grad.	Trend cf. doctorate	OR = -74% (CI: [-88%, -45%], p=0.1430)
100% non-animal: fungi-based	Education: undergrad. de-gree	Trend cf. doctorate	OR = -59% (CI: [-80%, -16%], p>0.9999)
**100% non-animal: algae-based**	**Education: high school**	**Effect cf. doctorate**	**OR = -90% (CI: [-96%, -75%], p=0.0003)**
**100% non-animal: algae-based**	**Education: below under-grad.**	**Effect cf. doctorate**	**OR = -78% (CI: [-90%, -54%], p=0.0221)**
100% non-animal: algae-based	Education: undergrad. de-gree	Trend cf. doctorate	OR = -67% (CI: [-84%, -32%], p=0.7150)

100% non-animal: fungi-based	Gender: male	Trend cf. female	OR = +127% (CI: [+27%, +304%], p>0.9999)
Insect-based	Pet/vet industry:	Trend cf. not in pet/vet industry	OR = +54% (CI: [+15%, +107%], p=0.9871)
Vegetarian	Income: high	Trend cf. low income	OR = +66% (CI: [+5%, +161%], p>0.9999)
**100% non-animal: plant-based**	**Region: Other European**	**Effect cf. UK residents**	**OR = +522% (CI: [+343%, +791%], p<0.0001)**
**100% non-animal: plant-based**	**Region: Oceania**	**Effect cf. UK residents**	**OR = +417% (CI: [+198%, +797%], p<0.0001)**
**Cultivated meat**	**Region: Other European**	**Effect cf. UK residents**	**OR = +124% (CI: [+61%, +211%], p=0.0004)**
100% non-animal: algae-based	Region: Other European	Trend cf. UK residents	OR = +76% (CI: [+4%, +196%], p>0.9999)
100% non-animal: algae-based	Region: Oceania	Trend cf. UK residents	OR = +181% (CI: [+41%, +458%], p=0.9018)
100% non-animal: fungi-based	Region: Oceania	Trend cf. UK residents	OR = +117% (CI: [+8%, +336%], p>0.9999)
Cultivated meat	Region: North America	Trend cf. UK residents	OR = +61% (CI: [+3%, +150%], p>0.9999)
Cultivated meat	Region: North America	Trend cf. UK residents	OR = +61% (CI: [+3%, +150%], p>0.9999)
Cultivated meat	Region: Oceania	Trend cf. UK residents	OR = +126% (CI: [+37%, +274%], p=0.4230)
No/minimal effect: Residence			
**DOG DEMOGRAPHICS**			
**100% non-animal: plant-based**	**Diet: raw meat**	**Effect cf. conventional meat**	**OR = -61% (CI: [-71%, -47%], p<0.0001)**
**100% non-animal: fungi-based**	**Diet: raw meat**	**Effect cf. conventional meat**	**OR = -63% (CI: [-76%, -43%], p=0.0014)**
**100% non-animal: algae-based**	**Diet: raw meat**	**Effect cf. conventional meat**	**OR = -59% (CI: [-73%, -38%], p=0.0105)**
**Vegetarian**	**Diet: raw meat**	**Effect cf. conventional meat**	**OR = -71% (CI: [-79%, -61%], p<0.0001)**
**Cultivated meat**	**Diet: raw meat**	**Effect cf. conventional meat**	**OR = -39% (CI: [-50%, -24%], p=0.0029)**
Insect-based	Diet: raw meat	Trend cf. conventional meat	OR = -28% (CI: [-44%, -7%], p>0.9999)
100% non-animal: plant-based	Medical diet	Trend cf. no medical diet	OR = +84% (CI: [+8%, +212%], p>0.9999)
Vegetarian	Medical diet	Trend cf. no medical diet	OR = +73% (CI: [+8%, +177%], p>0.9999)
100% non-animal: plant-based	Age: 5-9 years	Trend cf. 0-4 years	OR = +42% (CI: [+3%, +97%], p>0.9999)
100% non-animal: plant-based	Age: 10-14 years	Trend cf. 0-4 years	OR = +77% (CI: [+19%, +164%], p>0.9999)
100% non-animal: algae-based	Age: 5-9 years	Trend cf. 0-4 years	OR = +55% (CI: [+2%, +137%], p>0.9999)

100% non-animal: plant-based	Sex/neuter status: male, sexually intact	Trend cf. female, spayed	OR = -64% (CI: [-79%, -39%], p=0.0581)
100% non-animal: plant-based	Sex/neuter status: male, castrated	Trend cf. female, spayed	OR = -29% (CI: [-48%, -4%], p>0.9999)
Vegetarian	Sex/neuter status: female, sexually intact	Trend cf. female, spayed	OR = -60% (CI: [-78%, -30%], p=0.4100)
**Vegetarian**	**Sex/neuter status: male, sexually intact**	**Effect cf. female, spayed**	**OR = -62% (CI: [-76%, -40%], p=0.0130)**
Cultivated meat	Breed size: small	Trend cf. toy size	OR = -54% (CI: [-76%, -9%], p>0.9999)
No/minimal impact: Working dog status, exercise level			

Table S2b. Key statistics relating to RQ2b (essential characteristics of alternative dog foods) and Figure 11.

Note: Starred items in the first column relate to Figure S4 for human demographics, and Figure S5 for dog demographics. Effects are reported as odds ratios, including 95% confidence intervals (not corrected for multiple testing) and p-values corrected for multiple testing. Significant results (after multiple testing correction) are highlighted. P-values are not provided in cases of explorative analyses.

RQ2b: What are the essential characteristics of alternative dog foods?			
**Essential category / item***	**Variable item**	**Status cf. reference category**	**Statistics**
**HUMAN DEMOGRAPHICS**			
**Pet Focus I**	**Diet: reducetarian**	**Effect cf. omnivores**	**OR = +82% (CI: [+46%, +127%], p<0.0001)**
**Pet Focus I**	**Diet: pescatarian**	**Effect cf. omnivores**	**OR = +148% (CI: [+70%, +261%], p=0.0004)**
**Pet Focus I**	**Diet: vegetarian**	**Effect cf. omnivores**	**OR = +192% (CI: [+120%, +288%], p<0.0001)**
**Pet Focus I**	**Diet: vegan**	**Effect cf. omnivores**	**OR = +572% (CI: [+395%, +813%], p<0.0001)**
**Pet Focus II**	**Diet: reducetarian**	**Effect cf. omnivores**	**OR = +77% (CI: [+40%, +125%], p=0.0004)**
**Pet Focus II**	**Diet: pescatarian**	**Effect cf. omnivores**	**OR = +198% (CI: [+103%, +339%], p<0.0001)**
**Pet Focus II**	**Diet: vegetarian**	**Effect cf. omnivores**	**OR = +132% (CI: [+72%, +212%], p<0.0001)**
**Pet Focus II**	**Diet: vegan**	**Effect cf. omnivores**	**OR = +191% (CI: [+117%, +288%], p<0.0001)**
**Personal Focus**	**Diet: reducetarian**	**Effect cf. omnivores**	**OR = +58% (CI: [+24%, +101%], p=0.0343)**
Personal Focus	Diet: pescatarian	No trend cf. omnivores	OR = +37% (CI: [-11%, +110%], p>0.9999)
**Personal Focus**	**Diet: vegetarian**	**Effect cf. omnivores**	**OR = +113% (CI: [+58%, +188%], p=0.0002)**
**Personal Focus**	**Diet: vegan**	**Effect cf. omnivores**	**OR = +173% (CI: [+104%, +265%], p<0.0001)**
**Personal Values**	**Diet: reducetarian**	**Effect cf. omnivores**	**OR = +115% (CI: [+66%, +177%], p<0.0001)**
**Personal Values**	**Diet: pescatarian**	**Effect cf. omnivores**	**OR = +244% (CI: [+130%, +413%], p<0.0001)**
**Personal Values**	**Diet: vegetarian**	**Effect cf. omnivores**	**OR = +280% (CI: [+180%, +416%], p<0.0001)**
**Personal Values**	**Diet: vegan**	**Effect cf. omnivores**	**OR = +525% (CI: [+364%, +743%], p<0.0001)**
**Pet Focus I**	**Age: 40-49 years**	**Effect cf. 18-29 years**	**OR = -47% (CI: [-61%, -27%], p=0.0139)**
**Pet Focus I**	**Age: 50-59 years**	**Effect cf. 18-29 years**	**OR = -48% (CI: [-61%, -29%], p=0.0059)**
**Pet Focus I**	**Age: 60-69 years**	**Effect cf. 18-29 years**	**OR = -47% (CI: [-62%, -26%], p=0.0326)**
Pet Focus I	Age: 70+ years	Trend cf. 18-29 years	OR = -59% (CI: [-76%, -31%], p=0.1200)
Pet Focus II	Age: 40-49 years	Trend cf. 18-29 years	OR = -41% (CI: [-57%, -18%], p=0.2239)

Pet Focus II	Age: 50-59 years	Trend cf. 18-29 years	OR = -42% (CI: [-58%, -21%], p=0.1059)
Pet Focus II	Age: 60-69 years	Trend cf. 18-29 years	OR = -36% (CI: [-55%, -8%], p>0.9999)
Pet Focus II	Age: 70+ years	Trend cf. 18-29 years	OR = -45% (CI: [-68%, -5%], p>0.9999)
Personal Focus	Age: 30-39 years	Trend cf. 18-29 years	OR = -27% (CI: [-46%, -0.3%], p>0.9999)
**Personal Focus**	**Age: 40-49 years**	**Effect cf. 18-29 years**	**OR = -55% (CI: [-68%, -38%], p=0.0003)**
**Personal Focus**	**Age: 50-59 years**	**Effect cf. 18-29 years**	**OR = -55% (CI: [-67%, -37%], p=0.0003)**
**Personal Focus**	**Age: 60-69 years**	**Effect cf. 18-29 years**	**OR = -60% (CI: [-72%, -42%], p=0.0002)**
Personal Focus	Age: 70+ years	Trend cf. 18-29 years	OR = -63% (CI: [-79%, -33%], p=0.1274)
**Personal Values**	**Age: 40-49 years**	**Effect cf. 18-29 years**	**OR = -52% (CI: [-66%, -32%], p=0.0043)**
Personal Values	Age: 50-59 years	Trend cf. 18-29 years	OR = -36% (CI: [-54%, -12%], p=0.9529)
Personal Values	Age: 60-69 years	Trend cf. 18-29 years	OR = -38% (CI: [-57%, -10%], p>0.9999)
Personal Values	Age: 70+ years	Trend cf. 18-29 years	OR = -47% (CI: [-70%, -4%], p>0.9999)
Pet Focus I	Education: no high school	Trend cf. doctorate	OR = -86% (CI: [-96%, -59%], p=0.0656)
**Pet Focus I**	**Education: high school**	**Effect cf. doctorate**	**OR = -68% (CI: [-82%, -44%], p=0.0114)**
Pet Focus I	Education: below under-grad.	Trend cf. doctorate	OR = -60% (CI: [-77%, -32%], p=0.1250)
Pet Focus I	Education: undergrad. de-gree	Trend cf. doctorate	OR = -48% (CI: [-69%, -11%], p>0.9999)
Pet Focus II	Education: no high school	Trend cf. doctorate	OR = -78% (CI: [-93%, -28%], p>0.9999)
Pet Focus II	Education: high school	Trend cf. doctorate	OR = -53% (CI: [-73%, -18%], p>0.9999)
Personal Focus	Education: no high school	Trend cf. doctorate	OR = -81% (CI: [-94%, -38%], p=0.8833)
**Personal Focus**	**Education: high school**	**Effect cf. doctorate**	**OR = -70% (CI: [-83%, -47%], p=0.0061)**
Personal Focus	Education: below under-grad.	Trend cf. doctorate	OR = -54% (CI: [-73%, -22%], p=0.5789)
Personal Focus	Education: undergrad. de-gree	Trend cf. doctorate	OR = -46% (CI: [-68%, -7%], p>0.9999)
Personal Values	Education: no high school	Trend cf. doctorate	OR = -96% (CI: [-99%, -64%], p=0.5074)
**Personal Values**	**Education: high school**	**Effect cf. doctorate**	**OR = -70% (CI: [-83%, -47%], p=0.0076)**
Personal Values	Education: below under-grad.	Trend cf. doctorate	OR = -47% (CI: [-69%, -10%], p>0.9999)
**Pet Focus I**	**Region: Other European**	**Effect cf. UK residents**	**OR = +245% (CI: [+149%, +380%], p<0.0001)**
Pet Focus I	Region: Oceania	Trend cf. UK residents	OR = +140% (CI: [+47%, +291%], p=0.0685)
Pet Focus II	Region: Other European	Trend cf. UK residents	OR = +56% (CI: [+14%, +112%], p=0.7027)
**Personal Focus**	**Region: Other European**	**Effect cf. UK residents**	**OR = +84% (CI: [+35%, +152%], p=0.0182)**

**Personal Focus**	**Region: Oceania**	**Effect cf. UK residents**	**OR = +220% (CI: [+99%, +416%], p=0.0003)**
**Personal Values**	**Region: Other European**	**Effect cf. UK residents**	**OR = +143% (CI: [+78%, +232%], p<0.0001)**
**Personal Values**	**Region: Oceania**	**Effect cf. UK residents**	**OR = +228% (CI: [+102%, +431%], p=0.0003)**
Personal Focus	Pet/vet industry:	Trend cf. not in pet/vet industry	OR = -27% (CI: [-43%, -5%], p>0.9999)
Personal Values	Pet/vet industry:	No trend cf. not in pet/vet industry	OR = +23% (CI: [-4%, +58%], p>0.9999)
Pet Focus I	Income: high	Trend cf. low income	OR = +45% (CI: [+2%, +105%], p>0.9999)
Personal Focus	Gender: male	No trend cf. females	OR = +31% (CI: [-13%, +95%], p>0.9999)
Personal focus: social/cultural*	Gender: male	Explorative tendency cf. females	OR = +92% (CI: [-47%, +590%])
No/minimal effect: Residence			
**DOG DEMOGRAPHICS**			
**Pet Focus I**	**Diet: raw meat**	**Effect cf. conventional meat**	**OR = -57% (CI: [-64%, -48%], p<0.0001)**
**Pet Focus II**	**Diet: raw meat**	**Effect cf. conventional meat**	**OR = -34% (CI: [-46%, -19%], p=0.0094)**
**Personal Focus**	**Diet: raw meat**	**Effect cf. conventional meat**	**OR = -53% (CI: [-62%, -42%], p<0.0001)**
**Personal Values**	**Diet: raw meat**	**Effect cf. conventional meat**	**OR = -42% (CI: [-52%, -28%], p<0.0001)**
Pet Focus I	Age: 10-14 years	Trend cf. 0-4 years	OR = +58% (CI: [+21%, +108%], p=0.1340)
**Personal Values**	**Age: 5-9 years**	**Effect cf. 0-4 years**	**OR = +55% (CI: [+23%, +95%], p=0.0322)**
Personal Values	Age: 10-14 years	Trend cf. 0-4 years	OR = +59% (CI: [+18%, +114%], p=0.3249)
Pet Focus I	Sex/neuter status: male, sexually intact	Trend cf. female, spayed	OR = -31% (CI: [-49%, -7%], p>0.9999)
Pet Focus II	Sex/neuter status: female, sexually intact	Trend cf. female, spayed	OR = -35% (CI: [-56%, -4%], p>0.9999)
Pet Focus II	Sex/neuter status: male, castrated	Trend cf. female, spayed	OR = -23% (CI: [-38%, -4%], p>0.9999)
Pet Focus II: reputation*	Sex/neuter status: female, sexually intact	Explorative tendency cf. female, spayed	OR = -58% (CI: [-78%, -21%])
Pet Focus II: reputation*	Sex/neuter status: male, sexually intact	Explorative tendency cf. female, spayed	OR = -37% (CI: [-59%, -1%])
Personal Values	Sex/neuter status: male, sexually intact	Trend cf. female, spayed	OR = -37% (CI: [-55%, -12%], p>0.9999)
No/minimal effect: Working dog, exercise level, medical diet, breed size			

Table S3. Key statistics relating to RQ3 (information sources about dog diets) and Figure 13 from the main paper.

Note: Effects are reported as odds ratios, including 95% confidence intervals (not corrected for multiple testing) and p-values corrected for multiple testing. Significant results (after multiple testing correction) are highlighted.

RQ3: Where do dog guardians source information about dog diets from?			
**Source category**	**Variable item**	**Status cf. reference category**	**Statistics**
**HUMAN DEMOGRAPHICS**			
Product-Specific	Diet: vegan	Trend cf. omnivores	OR = +25% (CI: [+1%, +54%], p>0.9999)
Vet/Pet Care	Diet: pescatarian	Trend cf. omnivores	OR = +49% (CI: [+3%, +117%], p>0.9999)
Vet/Pet Care	Diet: vegan	Trend cf. omnivores	OR = -29% (CI: [-43%, -12%], p=0.2604)
**Media/Literature**	**Diet: vegan**	**Effect cf. omnivores**	**OR = +137% (CI: [+91%, +194%], p<0.0001)**
Media/Literature	Diet: vegetarian	Trend cf. omnivores	OR = +42% (CI: [+8%, +87%], p>0.9999)
Product-Specific	Age: 40-49 years	Trend cf. 18-29 years	OR = -30% (CI: [-47%, -7%], p>0.9999)
Product-Specific	Age: 50-59 years	Trend cf. 18-29 years	OR = -29% (CI: [-46%, -6%], p>0.9999)
**Product-Specific**	**Age: 60-69 years**	**Effect cf. 18-29 years**	**OR = -45% (CI: [-60%, -25%], p=0.0260)**
**Product-Specific**	**Age: 70+ years**	**Effect cf. 18-29 years**	**OR = -68% (CI: [-80%, -48%], p=0.0005)**
Media/Literature	Age: 40-49 years	Trend cf. 18-29 years	OR = -35% (CI: [-51%, -14%], p=0.4007)
Media/Literature	Age: 60-69 years	Trend cf. 18-29 years	OR = -39% (CI: [-55%, -17%], p=0.3333)
Media/Literature	Age: 70+ years	Trend cf. 18-29 years	OR = -54% (CI: [-71%, -25%], p=0.2971)
**Social Media**	**Age: 70+ years**	**Effect cf. 18-29 years**	**OR = -70% (CI: [-84%, -45%], p=0.0230)**
Vet/Pet Care	Age: 30-39 years	Trend cf. 18-29 years	OR = -37% (CI: [-52%, -17%], p=0.1785)
Vet/Pet Care	Age: 40-49 years	Trend cf. 18-29 years	OR = -40% (CI: [-54%, -21%], p=0.0560)
**Vet/Pet Care**	**Age: 50-59 years**	**Effect cf. 18-29 years**	**OR = -52% (CI: [-64%, -37%], p<0.0001)**
**Vet/Pet Care**	**Age: 60-69 years**	**Effect cf. 18-29 years**	**OR = -50% (CI: [-63%, -32%], p=0.0023)**
Product-Specific	Education: high school	Trend cf. doctorate	OR = -50% (CI: [-69%, -19%], p=0.8701)
**Vet/Pet Care**	**Education: no high school**	**Effect cf. doctorate**	**OR = -85% (CI: [-94%, -63%], p=0.0095)**
Vet/Pet Care	Education: high school	Trend cf. doctorate	OR = -42% (CI: [-64%, -5%], p>0.9999)
Media/Literature	Education: no high school	Trend cf. doctorate	OR = -78% (CI: [-91%, -49%], p=0.0805)

**Media/Literature**	**Education: high school**	**Effect cf. doctorate**	**OR = -67% (CI: [-80%, -45%], p=0.0041)**
Media/Literature	Education: below undergrad.	Trend cf. doctorate	OR = -57% (CI: [-74%, -28%], p=0.1975)
Media/Literature	Education: undergrad. Degree	Trend cf. doctorate	OR = -51% (CI: [-70%, -19%], p=0.8701)
**Media/Literature**	**Pet/vet industry**	**Effect cf. not in pet/vet industry**	**OR = +53% (CI: [+23%, +91%], p=0.0242)**
Social Media	Pet/vet industry	Trend cf. not in pet/vet industry	OR = -31% (CI: [-46%, -13%], p=0.2604)
**Media/Literature**	**Region: Other European**	**Effect cf. UK residents**	**OR = +72% (CI: [+35%, +120%], p=0.0030)**
**Media/Literature**	**Region: North American**	**Effect cf. UK residents**	**OR = +141% (CI: [+64%, +254%], p=0.0016)**
Media/Literature	Region: Oceania	Trend cf. UK residents	OR = +72% (CI: [+14%, +159%], p>0.9999)
Media/Literature	Gender: male	No trend cf. females	OR = +34% (CI: [-3%, +85%], p>0.9999)
Social Media	Gender: male	Trend cf. females	OR = -41% (CI: [-59%, -15%], p=0.6890)
No/minimal impact: Income, residence			
**DOG DEMOGRAPHICS**			
Product-Specific	Diet: raw meat	Trend cf. conventional meat	OR = -23% (CI: [-35%, -8%], p=0.7351)
**Vet/Pet Care**	**Diet: raw meat**	**Effect cf. conventional meat**	**OR = -39% (CI: [-49%, -27%], p<0.0001)**
**Vet/Pet Care**	**Diet: vegan**	**Effect cf. conventional meat**	**OR = -61% (CI: [-69%, -49%], p<0.0001)**
**Media/Literature**	**Diet: raw meat**	**Effect cf. conventional meat**	**OR = +46% (CI: [+22%, +74%], p=0.0066)**
**Media/Literature**	**Diet: vegan**	**Effect cf. conventional meat**	**OR = +165% (CI: [+104%, +243%], p<0.0001)**
**Social Media**	**Diet: raw meat**	**Effect cf. conventional meat**	**OR = +269% (CI: [+204%, +347%], p<0.0001)**
**Social Media**	**Diet: vegan**	**Effect cf. conventional meat**	**OR = +141% (CI: [+86%, +212%], p<0.0001)**
**Vet/Pet Care**	**Medical diet**	**Effect cf. no medical diet**	**OR = +237% (CI: [+118%, +419%], p<0.0001)**
**Product-Specific**	**Sex/neuter status: female, sexually intact**	**Effect cf. female, spayed**	**OR = -51% (CI: [-64%, -32%], p=0.0021)**
Product-Specific	Sex/neuter status: male, sexually intact	Trend cf. female, spayed	OR = -35% (CI: [-50%, -16%], p=0.2035)
Social Media	Breed size: small	Trend cf. toy size	OR = +91% (CI: [+2%, +259%], p>0.9999)
Social Media	Breed size: medium	Trend cf. toy size	OR = +104% (CI: [+10%, +278%], p>0.9999)
No/minimal impact: Dog age, working dog, exercise level			
